# Interface-Controlled Structural Behaviour of 3D Printed Concrete: Lessons from Masonry for Anisotropy, Print-Path Design and Standardisation

**DOI:** 10.3390/ma19173688

**Published:** 2026-08-30

**Authors:** Ali Mardani, Mohammad Hematibahar, Selin Özteber, Qais Abdulrahman Ali Qais, Abbas Abdulhussein Abd Noor, Tesfaldet Hadgembes Gebre, Ahmed Elsheikh

**Affiliations:** 1 Department of Civil Engineering, Bursa Uludag University, 16059 Nilufer-Bursa, Türkiye; ozteberselin@uludag.edu.tr; 2 Department of Architecture, Restoration and Design, RUDN University, Moscow 117198, Russia; eng.m.hematibahar1994@gmail.com (M.H.); tesfaldethg@gmail.com (T.H.G.); 3 Department of Construction Technologies and Structural Materials, Peoples’ Friendship University of Russia (RUDN), Moscow 117198, Russia; qaiseng@gmail.com; 4 Civil Engineering Department, Al-Muthanna University, Samawah 66001, Al-Muthanna, Iraq; abbas652002@mu.edu.iq; 5 College of Aerospace and Civil Engineering, Harbin Engineering University, Harbin 150001, China; ahmed.elshiekh19@feng.bu.edu.eg

**Keywords:** 3D printed concrete, masonry, interlayer bond, anisotropy, print-path design, standardization

## Abstract

The structural application of 3D printed concrete (3DPC) is still constrained by the difficulty of qualifying a layered, process-dependent material using standards developed mainly for cast concrete and conventional masonry. This review examines 3DPC through masonry construction to clarify how interface-controlled behaviour should be interpreted, tested and standardised. The comparison is not based on material similarity, but on the shared structural role of joints, interfaces and assemblage-level load transfer. Most previous reviews mainly discuss printability, mixture design, material development or general mechanical performance. This review instead places the printed interface at the centre of structural qualification, using masonry only as a reference for interpreting joint-controlled load transfer. The review shows that compressive strength alone is insufficient for structural qualification, since printed elements may fail through interlayer debonding, direction-dependent cracking, filament instability, reinforcement discontinuity or connection weakness. Flexural, shear, cyclic and seismic responses are particularly sensitive to interlayer quality, loading orientation and print-path geometry. Existing concrete, mortar and masonry standards remain useful references, but require 3DPC-specific reporting and testing. Accordingly, a tiered qualification route is proposed, progressing from fresh-state characterisation to interface-dominated testing and structural-scale validation.

## 1. Introduction

Three-dimensional printed concrete (3DPC) has moved from an experimental extrusion technique to one of the most active research areas in digital construction. Its appeal is evident: cementitious materials can be deposited layer by layer without conventional formwork, allowing faster production, lower labour demand, reduced formwork-related cost and greater geometric freedom than traditional casting methods [1,2,3,4]. However, the structural use of 3DPC is not determined only by whether a mixture can be pumped and extruded. A printed element is a layered cementitious body whose performance depends on how fresh filaments are deposited, supported, bonded and hardened. For this reason, the central challenge is no longer simply the production of printable concrete, but the qualification of printed concrete as a reliable structural material.

This challenge becomes clearer when 3DPC is examined through the lens of masonry construction. Masonry and 3DPC differ in age, material composition and production method, yet both systems are governed by interfaces. Masonry is not merely a stack of bricks or blocks, and 3DPC is not merely conventional concrete placed by a robotic nozzle. In masonry, the load path passes through units, mortar joints and contact regions; therefore, compressive, tensile, flexural and shear resistance depend on unit strength, mortar quality, bed-joint orientation, head-joint continuity, bond pattern, workmanship and confinement. The joint is not a secondary feature but one of the controlling elements of masonry behaviour. In 3DPC, a comparable structural issue appears in a different physical form. Each deposited filament creates a potential interface. Whether adjacent layers act as a continuous body or as partially bonded laminates depends on surface moisture, thixotropic rebuilding, early hydration, printing speed, nozzle pressure, interlayer time interval, surface roughness and local void formation [5,6,7,8,9,10,11]. The mechanism by which this occurs is examined in detail in Section 5.

The analogy between masonry and 3DPC is useful, but it must be handled carefully. In masonry, the interface is intentionally formed by placing mortar between hardened or semi-hardened units. It is visible, geometrically defined and already embedded in design philosophy through unit, prism, wallet and wall-level testing. In 3DPC, the interface is created by the printing process itself. It may not be visually distinct, and its quality can vary within the same element depending on deposition sequence, environmental exposure, rest time, filament deformation, surface moisture loss and curing conditions. Masonry can therefore provide a mature reference for treating joints and assemblages as structural variables, but masonry standards cannot be transferred directly to 3DPC without considering the different origins and variability of printed interlayers.

A fundamental difficulty in 3DPC is the conflict between printability and interlayer bonding. During pumping and extrusion, the mixture must be sufficiently flowable to pass through the delivery system without blockage, segregation or tearing. Immediately after deposition, however, the same material must develop enough yield stress and structural build-up to retain its geometry and support subsequent layers. This requirement creates a narrow rheological window. If the mixture remains too fluid, the printed filament may spread, deform or collapse under the weight of upper layers. If the mixture stiffens too rapidly, the lower layer may lose surface moisture and bonding ability before the next layer is deposited, increasing the likelihood of cold-joint formation and reduced interlayer bond strength [8,9,10,11,12,13]. Thus, the fresh-state behaviour of 3DPC is not only a construction-stage property; it directly governs hardened structural performance.

Interlayer bond controls the transition from material strength to element strength. Cast concrete is usually confined by formwork and may be compacted by vibration or self-consolidation, which helps reduce large interfacial discontinuities. In contrast, 3DPC is deposited without formwork and generally without vibration. The printed body may therefore contain aligned pores, incomplete contact zones and filament boundaries parallel to the print path. These features can reduce tensile and flexural resistance, influence crack initiation and propagation, and produce different strength values depending on loading direction and specimen extraction orientation [5,9,14,15,16]. A compressive strength measured on a printed cube or saw-cut prism may not represent the behaviour of a printed wall containing corners, restarts, path changes, layer-wise defects or reinforcement discontinuities. This is one of the reasons why direct use of conventional concrete tests can be misleading for structural qualification of 3DPC.

A related but distinct interface phenomenon arises when 3D-printed elements are used as permanent formwork bonded to post-cast concrete, rather than as fully printed structural members. Recent work on 3D-printed engineered cementitious composite (ECC) formwork shows that this print-to-cast interfacial bond is governed by the same class of process variables—single-layer printing height, fibre content and aggregate replacement ratio—with moderate fibre content and printing height improving bond strength while recycled sand addition reduces it [17]. This reinforces, from a different interface configuration, the central argument that interfacial performance in 3DPC is process-dependent rather than mixture-dependent alone.

The same issue appears in reinforcement and mixture design. Fibres, supplementary cementitious materials and chemical admixtures can improve selected properties of 3DPC, but their effects are not independent of the printing process. Fibres may improve flexural strength, crack bridging and dimensional stability; however, their efficiency depends on fibre type, aspect ratio, dosage, surface condition, orientation and the direction of the crack plane [14,15,16,18]. Mineral additions such as fly ash, silica fume and blast furnace slag can modify rheology, structural build-up, shrinkage and environmental impact, but an improvement in one property can be accompanied by a penalty in another. Fly ash may improve workability and contribute to later-age performance, while insufficient early stiffness may be unfavourable for buildability. Silica fume can increase cohesion and structural build-up, but its effect on flexural response and shrinkage depends on dosage and fibre interaction. High-volume blast furnace slag can reduce carbon emissions and alter rheological resistance, but the mixture may require additional adjustment to maintain extrusion quality and early-age stability [12,13,19,20]. Therefore, 3DPC cannot be evaluated through mix composition or compressive strength alone. The material design, rheological state, print parameters, interlayer condition and structural response must be interpreted together.

Reported effects of fibre and mineral-addition content are not always consistent across studies, and this inconsistency is itself informative. For example, some studies report that increasing fibre dosage monotonically improves flexural and crack-bridging performance [14,15,16,18], while others report an optimum fibre content beyond which workability loss and poor dispersion offset the mechanical gain. This discrepancy is best explained not by contradictory material behaviour, but by differences in mixing sequence, printing speed and nozzle geometry across studies—parameters that are inconsistently reported and therefore cannot be normalised for direct comparison. This illustrates a recurring limitation of the current literature: mechanical outcomes are frequently reported without the process parameters needed to reconcile conflicting results.

The lack of mature standards remains one of the main barriers to broader structural use of 3DPC. Conventional concrete standards provide useful baseline information, but they do not sufficiently describe filament geometry, print direction, interlayer interval, surface moisture, extraction location, cold-joint risk or direction-dependent strength. Masonry standards are informative because they already recognise the importance of unit–joint interaction and assemblage behaviour; however, they are based on deliberately formed mortar joints rather than process-generated cementitious interfaces. Recent standardisation activities confirm the importance of this gap. ACI Committee 564 addresses additive manufacturing with cementitious materials, and NIST has emphasised measurement science for performance-based standards for reinforced 3D printed concrete structures. RILEM TC 304-ADC interlaboratory studies have shown that test results remain difficult to compare unless printing procedure, specimen preparation, extraction direction and loading configuration are clearly defined [21,22,23,24,25].

Previous reviews on 3DPC have provided valuable accounts of printable mixtures, rheological requirements, buildability, reinforcement strategies, durability and broad standardisation needs. These reviews are essential for understanding material development, but they generally treat anisotropy and interlayer bonding as one property among several, rather than as the organising structural principle. As a result, the structural meaning of the printed interface has not always been separated clearly from general material performance, and the knowledge gap this creates is the absence of a structural, interface-first qualification logic for 3DPC.

The present review addresses this gap by examining 3DPC through masonry construction—not to classify printed concrete as masonry, but to use masonry as a mature, code-recognised reference for joint-controlled load transfer, anisotropy and assemblage-level testing. The novelty of the review lies in this structural reading of 3DPC: the printed interlayer is treated not as a production defect or a secondary material feature, but as a load-transfer region that must be reported, tested and qualified. This distinguishes the present work from earlier 3DPC reviews, which do not develop this direct analogy with an established structural testing hierarchy. On this basis, the review connects fresh-state behaviour, interlayer bond, print-path geometry, direction-dependent mechanical response, reinforcement continuity and standardisation into a single qualification route for printed structural elements.

## 2. Literature Basis and Scope of the Review

**Figure 1:**
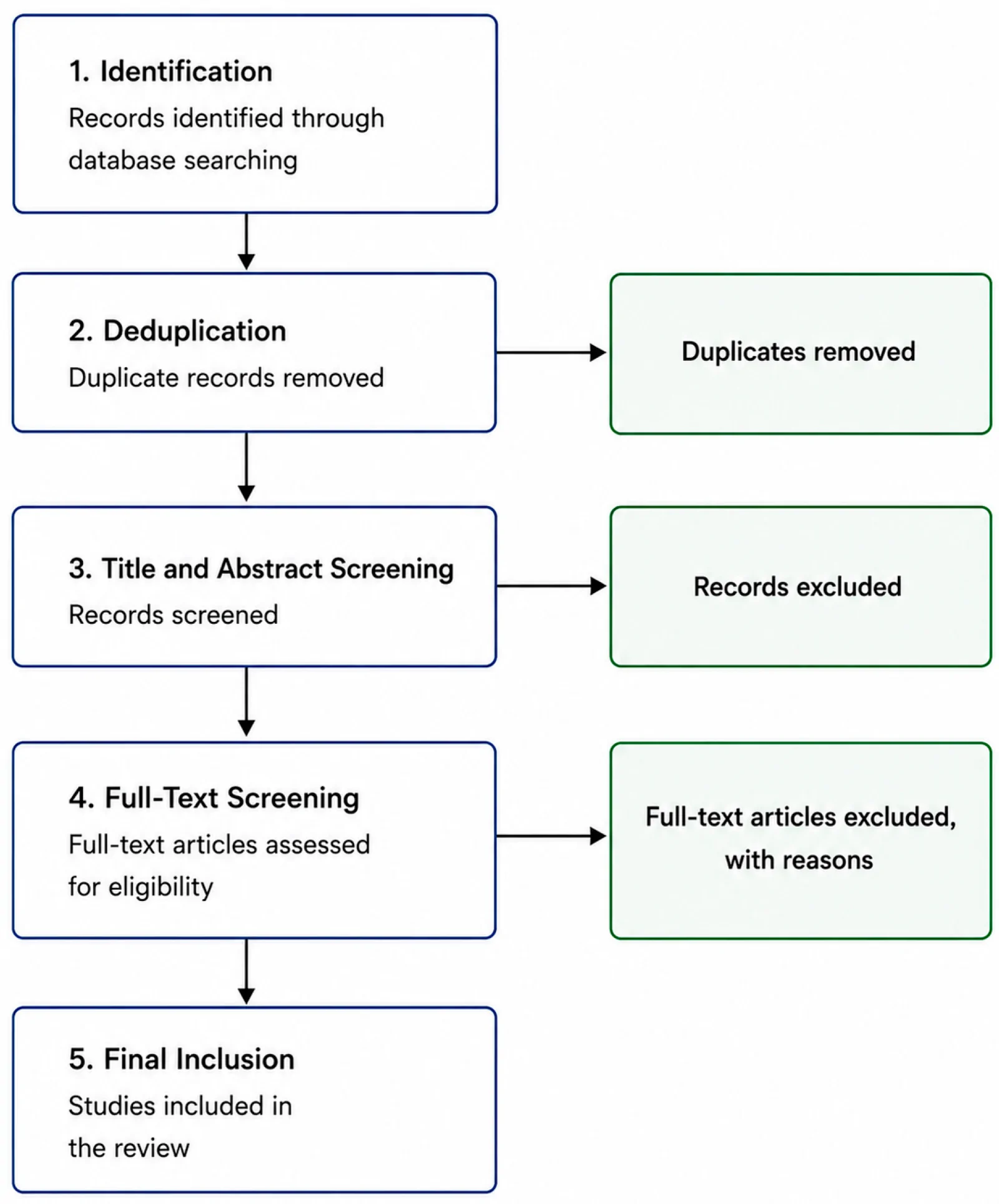
Flow diagram of the literature identification, duplicate removal, screening, eligibility assessment, and final inclusion process adopted in this review.

A structured search was carried out in Scopus, Web of Science and Google Scholar to identify studies relevant to interface-controlled structural behaviour in 3D printed concrete (3DPC). Search terms combined “3D printed concrete”, “3D concrete printing”, “interlayer bond”, “anisotropy”, “print-path”, “masonry”, “bed-joint” and “structural qualification” in various combinations, supplemented by citation tracking of key papers identified through this search. The search covered publications from 2007, when structural 3DPC research began to appear in the literature, through 2026, together with foundational masonry and seismic-engineering references predating this period where they were needed to establish testing and standardisation concepts. Titles and abstracts were first screened for relevance to interface-controlled structural behaviour; the full texts of potentially relevant records were then reviewed against the inclusion criteria described below. The literature identification, screening and inclusion process adopted in this review is presented in Figure 1. The complete list of studies included in the review is provided in Table S1 (Supplementary Materials).

This review is limited to studies that help explain how printed layers, interfaces and loading direction affect the structural behaviour of 3DPC. The masonry literature was used only where it provides a useful reference for joint-controlled behaviour, bond pattern, assemblage testing or standardisation logic. Durability, permeability, fire resistance and life-cycle issues were not treated as independent topics; they were considered only when they influenced interlayer integrity, cracking, deformation capacity or test development.

The literature basis of the review was selected to support this structural focus. The literature was selected in three steps. First, 3DPC studies dealing with printability, buildability, rheology, interlayer bond, anisotropy, reinforcement, mechanical testing and standardisation were examined. Second, masonry studies and standards were included when they dealt with joints, bed-joint orientation, bond pattern, prism or wallet testing, shear transfer or wall-level behaviour. Third, papers on durability, sustainability or mixture modification were retained only when their findings had a direct bearing on structural response, such as shrinkage-related cracking, surface moisture loss, pore alignment, fibre orientation or interface degradation. Papers dealing only with material optimisation, environmental assessment or architectural application were not emphasised unless they clarified one of these structural mechanisms.

The review then follows four linked themes. These are: the material and process variables that create anisotropy in 3DPC; the extent to which this anisotropy can be interpreted using masonry concepts such as joints, bed-joint orientation and bond pattern; the need to move from material-level tests toward element- and wall-level qualification; and the limited but useful transferability of existing concrete and masonry standards. This organisation separates the present review from broader 3DPC reviews by keeping the discussion centred on interface-controlled structural behaviour.

The studies included in Table 1 were selected based on their direct relevance to the main structural themes of this review, including interlayer bonding, anisotropy, mechanical response, reinforcement, printing-process effects, and standardisation. Studies were prioritised when they provided information relevant to the interpretation of interface-controlled structural behaviour. Table 1 summarises selected studies and standardisation activities that directly support the interface-focused structural scope of this review. The table is not intended to list all 3DPC publications. Instead, it identifies papers and guidance documents that directly inform the structural argument of this review. These sources explain how printed layers are formed, how interfaces affect load transfer, how anisotropy changes mechanical response and why test results remain difficult to compare without clear reporting of printing and specimen preparation. The final column of the table explains how each source supports, limits or refines the masonry–3DPC comparison.

**Figure 2:**
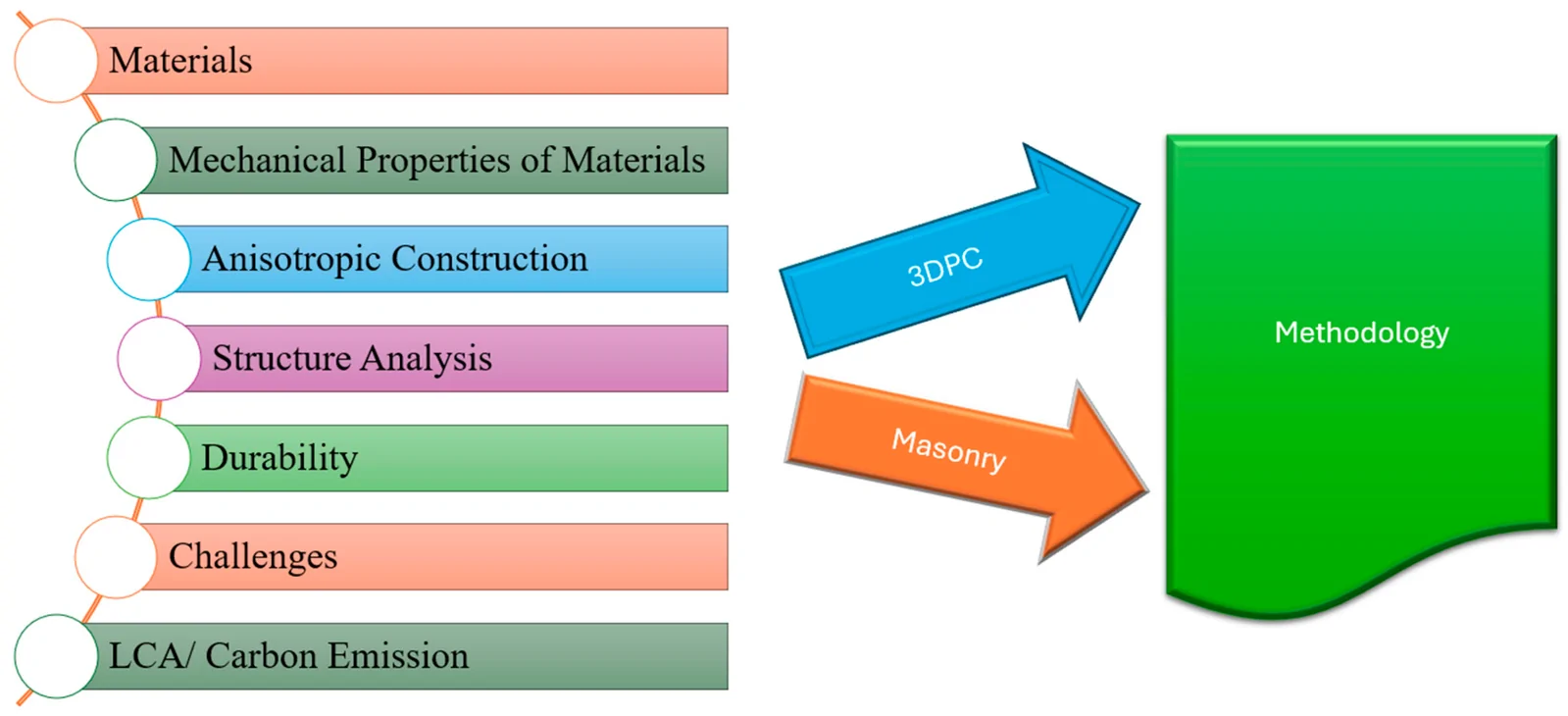
Review route linking material selection, fresh-state requirements, interlayer bond, anisotropic mechanical response, structural behaviour and standardisation.

The review route adopted in this paper is shown in Figure 2. The route begins with constituent materials and fresh-state requirements because these variables determine whether stable and bondable layers can be produced. It then proceeds to interlayer bond and anisotropic mechanical response, where the influence of print direction, interlayer quality and loading orientation becomes visible. The next level is structural response, including compressive, flexural and shear behaviour, reinforcement continuity and dynamic loading. Finally, these observations are connected to standardisation, where existing concrete and masonry tests are assessed according to whether they can capture the layered nature of printed concrete.

## 3. Constituent Materials and Process Variables Controlling Structural Behaviour

The structural comparison between masonry construction and 3D printed concrete (3DPC) should begin with the way each system transforms constituent materials into a load-bearing assemblage. Masonry includes fired clay bricks, unfired earth bricks, compressed earth blocks, cob, mortar, grout, lime- and cement-stabilised units, recycled aggregate blocks and fibre-reinforced earthen or cementitious units [26,27,28,29,30,31,32,33,34]. 3DPC includes ordinary Portland cement (OPC), calcium sulfoaluminate cement (CSA), magnesium potassium phosphate cement (MPC), geopolymer binders, printable ECC/UHPC-based mixtures, fine aggregates, mineral fillers, superplasticisers, viscosity-modifying admixtures and fibres [8,9,10,11,12,13,14,15,16,18,35,36,37,38,39,40,41,42,43,44,45,46]. These material families cannot be compared through compressive strength alone. In masonry, strength is generated by the interaction between units and mortar joints. In 3DPC, strength is governed by the coupled action of mixture rheology, filament geometry, interlayer contact and time-dependent bond formation.

This distinction is important because material optimisation has different consequences in the two systems. In masonry, the unit is generally manufactured before wall construction, while the mortar joint is formed during laying. The final wall behaviour depends on unit strength, mortar stiffness, suction, joint thickness, bond pattern and workmanship. In 3DPC, the material is shaped and assembled at the same time. The deposited filament is simultaneously the fresh material, the construction unit and the surface onto which the next layer must bond. Thus, the printed interface is not a separately designed joint; it is a process-generated region whose quality depends on the state of the lower layer at the moment the upper layer is deposited.

The key material challenge in 3DPC is the balance between buildability and interlayer bonding. A mixture must be sufficiently flowable during pumping and extrusion, but sufficiently stiff immediately after deposition to resist self-weight, extrusion pressure and the weight of subsequent layers. When structural build-up is too slow, the printed element may suffer from filament spreading, shape loss, local buckling or collapse. When structural build-up is too rapid, the surface of the lower layer may lose moisture and deformability before the next layer arrives, reducing intimate contact and increasing the risk of cold-joint formation [9,10,11,12,13]. Therefore, the fresh-state behaviour of 3DPC is not merely a production requirement; it is one of the origins of hardened anisotropy.

A similar principle exists in masonry, although the physical mechanism is different. The bed joint and head joint define preferential planes for cracking, sliding, tensile separation and shear transfer. In printed concrete, preferential planes arise from filament boundaries, pore alignment, incomplete contact, surface drying and changes in hydration state across the interface. Thus, the masonry analogy is useful only when it is limited to interface-controlled load transfer. This makes process documentation in 3DPC as important as material composition.

The role of fibres also differs between masonry materials and 3DPC. In masonry units, fibres such as straw, polypropylene, glass, steel, banana or pineapple leaf fibres are generally used to improve crack resistance, toughness, dimensional stability or thermal performance of the unit itself [31,32,33,34,47,48,49,50,51]. In 3DPC, fibres may increase flexural strength and crack bridging, but they also modify rheology, pumpability, extrudability, filament stability and fibre orientation. Extrusion tends to align fibres along the print direction; therefore, an increase in fibre dosage does not necessarily improve interlayer tensile resistance if the fibres do not cross the critical crack plane. Excessive fibre content or inappropriate aspect ratio may increase flow resistance, disturb filament continuity or create weak regions at the interface [14,15,16,18]. For this reason, fibre reinforcement in 3DPC must be interpreted together with nozzle geometry, print direction and loading orientation.

Supplementary cementitious materials and fine fillers introduce a similar trade-off. Fly ash may improve workability through particle packing and a ball-bearing effect, but excessive replacement can reduce early-age stiffness and delay strength development, which may be unfavourable for buildability. Silica fume can increase cohesion, viscosity and structural build-up due to its high fineness and pozzolanic activity, but high contents may increase water demand and affect shrinkage behaviour. Blast furnace slag can reduce cement-related environmental burden and contribute to later-age strength, but its effect on early rheology and structural build-up depends on replacement level, particle characteristics and admixture compatibility [12,13,16,18]. These mechanisms show why printable mixtures cannot be selected only by sustainability potential or final compressive strength. A low-carbon binder is useful for structural 3DPC only if it also provides a printable rheological window, sufficient early stability and reliable interlayer bonding.

Read together, the studies compiled in Table 1 converge on a consistent pattern rather than a set of unrelated findings: printability, rheology and interlayer bond are repeatedly reported as interacting variables rather than independent material properties, and the standardisation efforts included in the table (ACI Committee 564, NIST, RILEM TC 304-ADC) confirm that reporting of print-process parameters remains inconsistent across studies. The main gap emerging from this body of work is therefore not a lack of individual findings, but the absence of a shared reporting framework that would allow these findings to be compared or combined.

Chemical admixtures further illustrate the process sensitivity of 3DPC. Superplasticisers reduce yield stress and improve flow, which is beneficial during pumping and extrusion. However, excessive dispersion may reduce early shape stability after deposition. Viscosity-modifying admixtures, nano-clay, silica fume and other fine additions can improve cohesion and buildability. However, they may also increase extrusion pressure and reduce the ability of the next layer to merge with the previous one if stiffening becomes too rapid [8,11,13]. Therefore, the most relevant parameter is not the isolated effect of an admixture on flowability, but its effect on the sequence of pumping, extrusion, deposition, rest time and interlayer bond development.

Table 2 summarises representative masonry material modifications relevant to the present comparison. The masonry material modifications compiled in the table show a recurring trade-off pattern: interventions that improve one property, such as crack bridging, cohesion or sustainability, tend to penalise another, such as workability, density or water demand. This mirrors the trade-off structure observed in 3DPC mixture design and supports the use of masonry material development as a conceptual, rather than literal, precedent for 3DPC mixture optimisation. The table shows that masonry materials have long been engineered through changes in soil–sand–clay proportion, fibre addition and stabilising constituents. This is important because 3DPC faces a similar design problem at a different scale: the improvement of one property may reduce another. Increased fibre content may improve crack bridging but reduce workability. Recycled or lightweight inclusions may reduce density but lower strength; very fine fillers may improve cohesion but increase water demand; and rapid structural build-up may improve buildability while reducing interlayer bonding.

The process variables specific to 3DPC are summarised in Table 3. These variables are included because they govern the transition from fresh mixture to structural element. Unlike masonry, where the unit and joint are usually produced in separate operations, 3DPC forms both the unit-like filament and the interface during a single continuous process. Consequently, print speed, nozzle geometry, layer height, interlayer time interval, surface moisture, structural build-up rate and specimen extraction direction must be reported together with the mixture design. Taken together, these variables point to a common conclusion: nearly every process parameter listed in Table 3 affects structural performance primarily through its influence on interlayer contact quality, rather than through the bulk cementitious matrix alone—which is why process parameters, and not mixture composition alone, must accompany any reported structural result.

**Figure 3:**
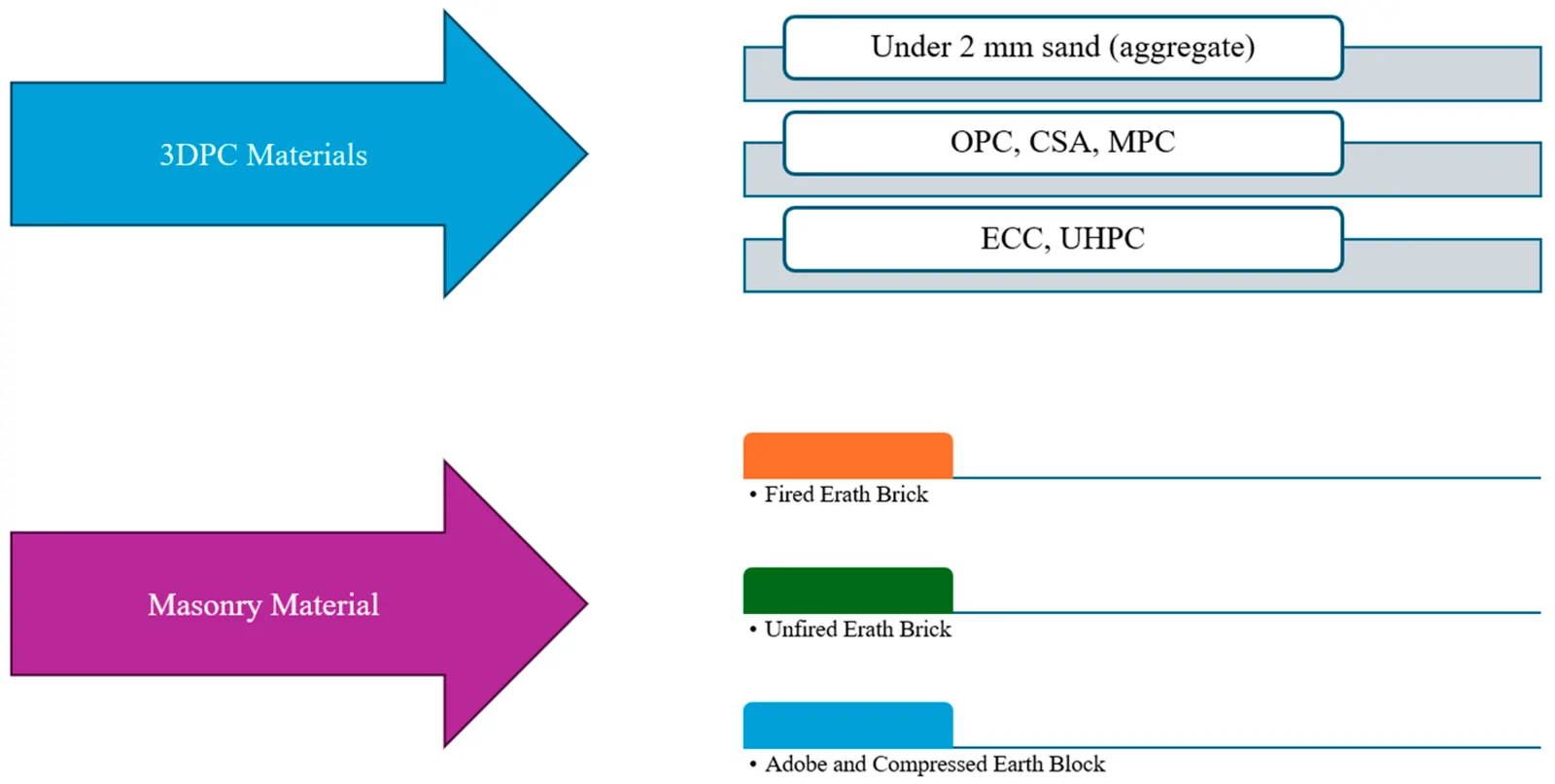
Material families and process variables governing interface-controlled behaviour in masonry construction and 3DPC.

Figure 3 summarises the material families and process variables that connect masonry and 3DPC within the scope of this review. Masonry is represented by unit-based materials and deliberately formed joints, whereas extrusion-based cementitious composites and process-generated interlayers represent 3DPC. The comparison is therefore not based on material identity, but on the way each system transfers load through interfaces.

## 4. Mechanical Properties: From Material Coupons to Assemblage Behaviour

Mechanical characterisation of masonry and 3D printed concrete (3DPC) requires a distinction between material indices and structural behaviour. Masonry units are commonly classified according to compressive strength; however, the design behaviour of masonry is not obtained from isolated units alone. The load-bearing response of a masonry wall depends on the combined action of units, mortar joints, bond pattern, joint orientation, confinement and workmanship. For this reason, prisms, wallets and wall specimens are more representative than individual bricks when the objective is to evaluate structural performance. The same logic applies to 3DPC. A printed cube, prism or core may provide a useful material index, but it cannot fully represent filament continuity, interlayer alignment, corner deposition, restart zones, print-path changes, reinforcement interruptions or wall-scale instability.

The direct comparison of masonry-unit strength and 3DPC material strength can therefore be misleading. Masonry brick classes may include compressive-strength levels of approximately 3.5–7.5 MPa for common wall applications, whereas selected 3DPC mixtures can reach considerably higher compressive-strength values under specific mixture and printing conditions [1,2,26,53]. These values should not be interpreted as evidence that 3DPC is structurally superior to masonry. They describe different levels of material response. A masonry wall may fail through joint crushing, bed-joint sliding, diagonal tension, rocking or unit splitting, even when the individual unit strength is adequate. Similarly, a printed wall may fail through interlayer debonding, tensile splitting along a pore-aligned region, local buckling of a filament, instability during construction or stress concentration at a restart location. This can happen even when the bulk cementitious matrix has high compressive strength.

The mechanism behind this difference is the scale transition from coupon to assemblage. In masonry, the unit and mortar joint have different stiffness, strength and deformation capacity. Under compression, the mortar may laterally deform and induce tensile stresses in the units; under shear or bending, cracks often follow the weaker joint or unit–mortar interface. In 3DPC, the interface is not a mortar joint. However, it may behave as a preferential plane if interlayer contact is incomplete, surface moisture is insufficient, pores are aligned or the lower layer has stiffened before the upper layer is deposited. Consequently, a printed material with high matrix strength may still show low tensile or flexural resistance across the interlayer region. This is particularly important because flexural and tensile failures are more sensitive to interfacial defects than compressive tests.

Table 4 presents representative masonry brick strength classes. These values are useful for unit classification, but they should not be used as direct structural analogues for printed concrete. Their value in this review is to show that masonry design already separates unit-level classification from assemblage-level performance. This distinction provides an important precedent for 3DPC, where printed specimens must be interpreted according to print direction, layer orientation, extraction location and loading configuration.

For 3DPC, mechanical properties are more strongly affected by production variables than in conventional cast specimens. The same cementitious matrix may show different compressive, tensile and flexural values when printed with different nozzle geometries, aggregate sizes, fibre contents, binder systems, printing speeds or interlayer intervals. Loading direction is also decisive. A specimen loaded perpendicular to the layers may activate a different failure path than a specimen loaded parallel or lateral to the printed filaments. Therefore, a single compressive-strength value cannot describe the mechanical performance of a printed structural element.

Table 5 summarises selected mechanical-property observations from 3DPC studies. The purpose of the table is not to rank mixtures by strength, but to show that the reported values are tied to printing and mixture variables. The same table also illustrates why 3DPC test methods must report process parameters together with mechanical results. Without information on nozzle size, print direction, interlayer interval, specimen extraction and loading orientation, data from different studies cannot be reliably compared.

The values compiled in Table 5 illustrate a broader pattern rather than a single trend: reported compressive strengths for 3DPC range from roughly 75–102 MPa in some studies to considerably lower or higher values in others, and these differences are not primarily due to disagreement about material behaviour. They largely reflect variation in nozzle geometry, binder chemistry, fibre content and specimen extraction direction, none of which are consistently reported across the cited studies. This makes direct cross-study comparison of absolute strength values unreliable and reinforces the more general limitation noted throughout this review: mechanical results in 3DPC cannot be meaningfully ranked or compared unless they are reported together with the process parameters that produced them.

The most important conclusion from these data is that strength in 3DPC is a process-dependent material property. In cast concrete, specimen geometry and curing history are also important, but the material is usually assumed to be comparatively continuous at the scale of standard tests. In 3DPC, the printing process creates structural features that are not incidental: filaments, interfaces, aligned pores, variable compaction, surface drying and restart zones. These features may dominate the failure mode, especially under flexure, tension, shear and dynamic loading.

This issue is particularly relevant for flexural behaviour. Flexural tests expose the weakness of the interlayer region more clearly than compressive tests because crack initiation is sensitive to tensile defects and weak planes. If the critical crack crosses several well-bonded filaments, the specimen may behave as a relatively continuous cementitious composite. If the crack follows a poorly bonded interface, the measured flexural strength may be governed by interlayer adhesion rather than matrix strength. Fibre reinforcement can improve crack bridging, but only when fibres are effectively oriented across the crack path. Since extrusion tends to align fibres along the print direction, the beneficial effect of fibres may be greater along the filament than across the interlayer region.

The same reasoning applies to shear and compression. In compression, printed specimens may fail by crushing of the matrix, splitting along interfaces or local instability of filaments depending on layer geometry and loading direction. In shear, the resistance may depend on cohesion across the interlayer, mechanical interlock created by filament surface roughness, normal stress across the interface and any reinforcement crossing the potential sliding plane. Masonry joints are filled with mortar and influenced by unit suction, joint thickness and workmanship; printed interfaces are controlled by rheology, surface moisture, open time, nozzle pressure and deposition history.

For this reason, mechanical qualification of 3DPC should move from isolated coupon testing toward a hierarchy of tests. Material coupons remain necessary for determining basic compressive, tensile and flexural indices. However, interlayer bond tests, direction-dependent flexural and shear tests, wall or panel tests, reinforcement-continuity assessment and specimens containing realistic print features such as corners, path changes and restarts should supplement them. The extraction direction, number of layers, position within the printed element, loading direction and curing condition should be reported as part of the mechanical result rather than as secondary information.

Long-term, time-dependent structural performance has received comparatively little attention in the 3DPC literature. Creep is reported to concentrate at the interlayer rather than in the bulk matrix, driven by weak interlayer bond under sustained loading [58]. Shrinkage-related cracking during curing has similarly been linked to interlayer condition [59]. Environmental exposure, particularly freeze–thaw cycling, is the most extensively documented long-term degradation mechanism: cracks preferentially initiate and propagate at interlayer regions, and vertically cored specimens crossing these interfaces degrade faster than horizontally cored specimens [60]. Fatigue behaviour specific to the printed interlayer remains comparatively unstudied and is a clear direction for future research. These findings support treating creep, shrinkage and environmental durability as interlayer-specific properties rather than properties inherited from cast-concrete practice.

This need is consistent with recent standardisation efforts. Interlaboratory studies conducted within RILEM TC 304-ADC have shown that mechanical results for 3DPC remain difficult to compare when laboratories use different mixtures, printers, specimen extraction procedures, curing regimes and test configurations [23,24,25]. These studies reinforce the central argument of this review: 3DPC cannot be qualified by transferring ordinary cast-concrete tests without modification. A reliable framework must connect mixture design, printing parameters, specimen preparation and structural response. Masonry provides a useful precedent because it already treats the assemblage, not only the unit, as the basis for structural evaluation.

Beyond compressive strength, systematic comparison between coupon- and assemblage-scale behaviour remains uneven across other mechanical properties, although targeted studies exist for several of them. The RILEM TC 304-ADC interlaboratory study [25] found that the modulus of elasticity is more strongly affected by interlayers than compressive strength: achieving a given modulus of elasticity required a higher compressive strength in printed specimens than in cast specimens, indicating that printed concrete is comparatively more compliant for a given strength level. The same study found that specimen scale itself matters: increasing specimen size from mortar-scale to concrete-scale generally reduced compressive strength slightly, consistent with the size effect long documented for cast concrete, although this effect was smaller than that associated with loading direction or cold joints. Direct and splitting tensile strength are addressed by the companion study [24]. Poisson’s ratio has also been shown to be direction-dependent: the study [61] found that printed specimens tested parallel and perpendicular to the print direction showed Poisson’s ratios up to 33% and 17% higher, respectively, than cast specimens, attributed to the oblate voids characteristic of the printed microstructure. Fracture energy is similarly interlayer-sensitive: the study [62] shows, using notched printed beams, that less energy is required to propagate a crack along an interlayer than through the bulk matrix, confirming the interlayer as the governing plane for crack propagation as well as for strength. However, these properties have been characterised mainly at the coupon scale, and their systematic transfer to assemblage-scale behaviour remains comparatively limited.

A direct scale-transition method has recently been proposed by adapting the masonry prism test to 3DPC [63] printed mortar and concrete prisms, which achieved strength-to-material ratios of 89% and 86%, respectively, closely matching the 88% ratio obtained from masonry prisms tested under the same protocol, and the surface strain distribution on the printed prisms matched that observed in full-scale wall tests. This suggests that masonry-derived prism testing can serve as a practical, cost-effective proxy for full-scale structural behaviour. Separately, full-scale compression tests on 3D-printed walls, compared against small coupons extracted from the same printed elements [64], confirmed that 3D printing reduces both stiffness and compressive strength relative to coupon-level expectations, reinforcing the need for validated scale-transition methods rather than direct extrapolation from coupon data.

## 5. Anisotropy as the Central Structural Problem

Anisotropy is one of the main reasons why masonry and 3D-printed concrete (3DPC) cannot be evaluated by a single material-strength value. In masonry, anisotropy arises from the coexistence of relatively stiff units and weaker mortar joints, the orientation of bed and head joints, the bond pattern, unit geometry and workmanship. These variables determine whether cracks pass through the units, follow the joints, develop along the unit–mortar interface or form diagonal failure paths under combined compression and shear [65,66,67,68,69]. In 3DPC, anisotropy originates from a different manufacturing mechanism. The printed element is built from filaments whose geometry, interlayer contact, pore distribution, local compaction, hydration state and fibre orientation are governed by the extrusion and deposition process [5,9,10,14,23,24,25]. Although the origins are different, the structural consequence is similar: tensile strength, shear resistance, flexural capacity and fracture behaviour cannot be represented reliably by a single direction-independent value.

The interlayer region is the most critical source of anisotropy in 3DPC. This weakness is not caused merely by the visual separation of printed layers. It is produced by changes in contact quality and microstructure at the boundary between two filaments. During the interval between successive layers, the lower filament may lose surface moisture, undergo thixotropic rebuilding and begin early hydration. These changes increase its resistance to deformation and reduce the ability of the upper layer to penetrate surface irregularities and form a continuous cementitious bridge. At the same time, entrapped air and printing-induced voids may remain aligned along the filament surface. The resulting interlayer zone can therefore contain incomplete contact areas, locally increased porosity and reduced cohesion. Under bending, tension or shear, such regions act as preferential paths for crack initiation and propagation [9,10,11,23,24,25].

This mechanism explains why flexural behaviour is often more sensitive to printing direction than compressive behaviour. In compression, load transfer may still occur through the filament body even when the interlayer is locally imperfect, although splitting or instability can develop if the weak planes are unfavourably oriented. In flexure and tension, however, the crack path is strongly affected by the weakest continuous region. If the tensile stress acts across a poorly bonded interface, failure may occur at lower stress than expected from the matrix strength. If the crack is forced to cross well-bonded filaments, the measured resistance may be higher. Therefore, the apparent flexural strength of 3DPC depends not only on mixture composition but also on layer orientation, interlayer interval, surface condition, extraction direction and loading configuration.

Masonry provides a useful reference for understanding this behaviour because masonry design has long recognised that joint orientation controls failure. A wall loaded normal to the bed joints, a panel subjected to out-of-plane bending, and a wallet tested under diagonal compression do not activate the same failure mechanism. Similarly, a 3DPC specimen loaded parallel, perpendicular or lateral to the printed layers may not develop the same crack path. The difference is that masonry joints are deliberately produced with mortar and their geometry is usually visible, whereas printed interlayers are formed during deposition and may be hidden within the hardened element. This makes the anisotropy of 3DPC more difficult to inspect and more dependent on process control.

Fibre reinforcement can reduce crack opening and improve post-cracking response, but it should not be treated as a universal solution for anisotropy. Fibres are effective when they bridge the expected crack plane and transfer stress across the opening crack. In extrusion-based 3DPC, however, the flow field inside the pump, hose and nozzle tends to align fibres along the print direction. This alignment may improve resistance along the filament, but it may provide limited benefit across the interlayer plane if few fibres cross the critical crack path. Fibre type, aspect ratio, dosage, surface condition and orientation therefore have to be interpreted together with nozzle geometry, print direction and loading direction [14,15,16,18]. Excessive fibre content may also increase flow resistance, disturb filament continuity and reduce print quality, which can indirectly worsen interlayer behaviour.

**Figure 4:**
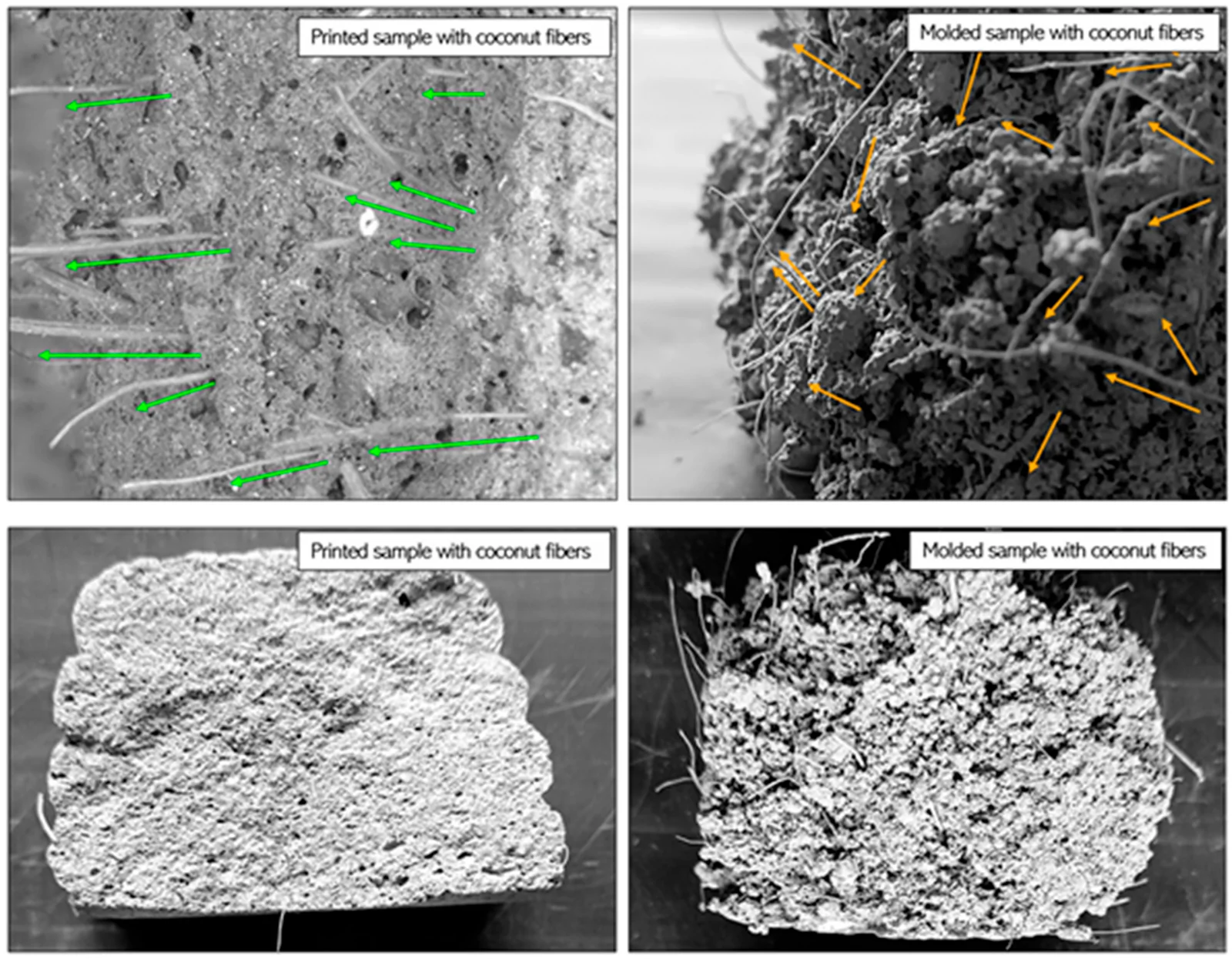
Fibre orientation and crack-bridging mechanism in coconut-fibre-reinforced foamed concrete (0.5–1 wt.% fibre content), comparing printed and cast (moulded) samples. Top row: cross-sections showing fibre alignment, with green arrows indicating fibre orientation in printed samples and orange arrows in cast samples; bottom row: corresponding sample cross-sections. Adapted from [70].

Figure 4 illustrates the importance of fibre orientation and crack bridging in printed and cast cementitious specimens. The key point is that reinforcement efficiency in 3DPC is controlled not only by fibre volume but also by the relationship between fibre alignment and the fracture plane.

The anisotropic behaviour of 3DPC also affects the interpretation of test results. A specimen cut from the same printed element may give different values depending on whether the loading path crosses the layers, follows the layers or acts within a filament. The number of interfaces included in the specimen, the distance from corners or restart points, the presence of surface defects and the curing condition may also affect the measured response. For this reason, mechanical properties of 3DPC should be reported together with specimen orientation, extraction position, number of layers, loading direction, interlayer interval and print-path information. Without these details, strength values from different studies may appear contradictory even when the underlying behaviour is consistent.

The central structural implication is that anisotropy must be treated as a design and qualification issue, not as a secondary material imperfection. For masonry, this principle is already reflected in the distinction between unit tests, prism tests, wallet tests and wall-scale tests. For 3DPC, an equivalent hierarchy is still developing. Direction-dependent compressive, flexural, tensile and shear tests should be linked to interlayer bond tests and wall-scale validation. Until such links are established, the use of direction-independent material properties for printed structural elements remains unsafe. A rational standardisation route should therefore define not only the test method but also the print orientation, specimen extraction procedure, interlayer configuration and loading direction associated with each reported property.

## 6. Print-Path Engineering and Interfacial Toughening

The interlayer region of 3D-printed concrete (3DPC) is often treated as an unavoidable weak plane, but it can also be modified through print-path engineering and interfacial toughening strategies. The basic principle is to prevent the crack from propagating along a straight and continuous layer boundary. If the interface remains planar, poorly bonded and aligned with the tensile stress field, crack growth requires comparatively low energy. If the interface is geometrically interlocked, periodically interrupted or forced to change direction, crack propagation becomes more difficult because the fracture path must twist, branch, bridge or pass through the filament body.

**Figure 5:**
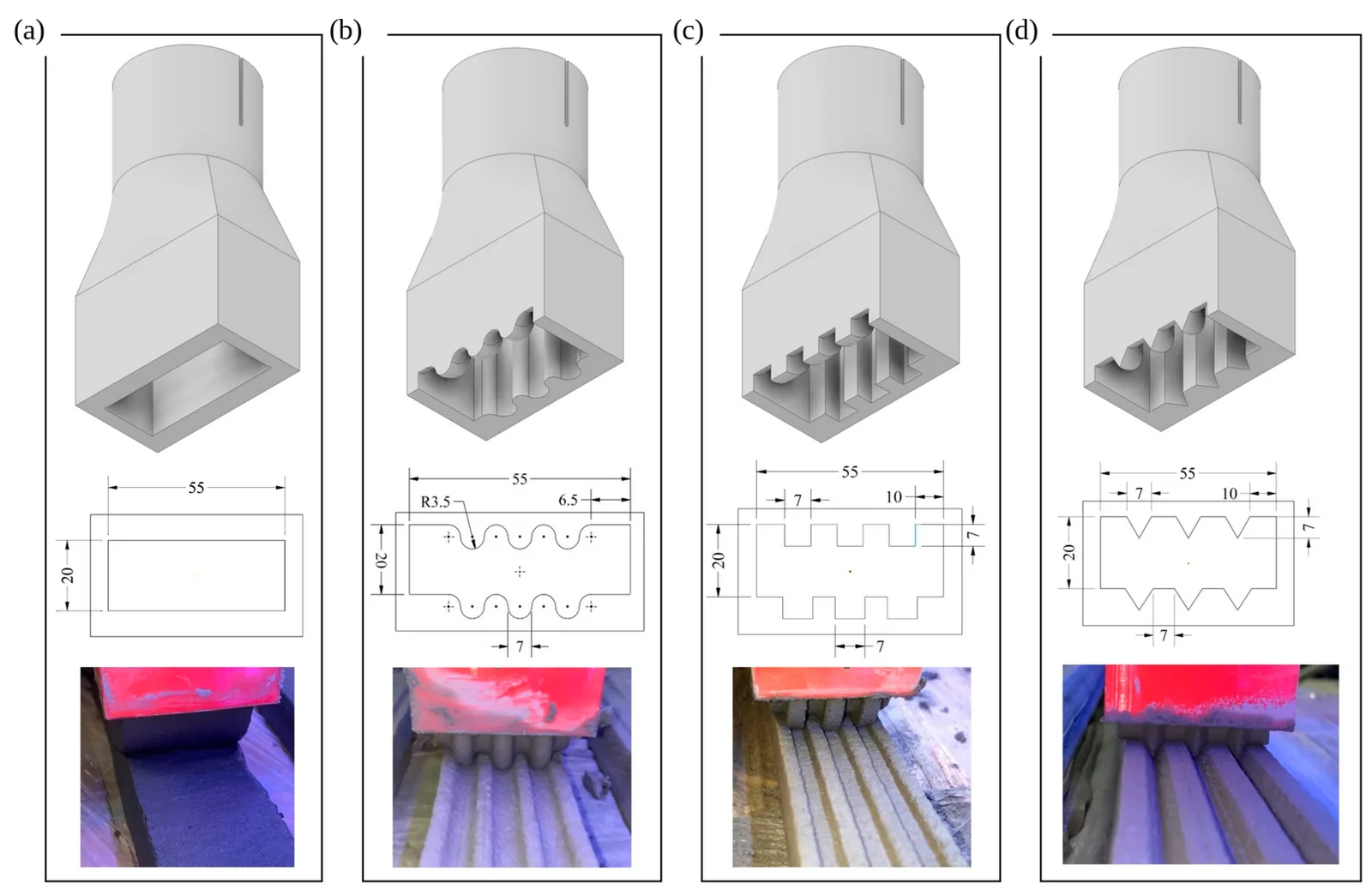

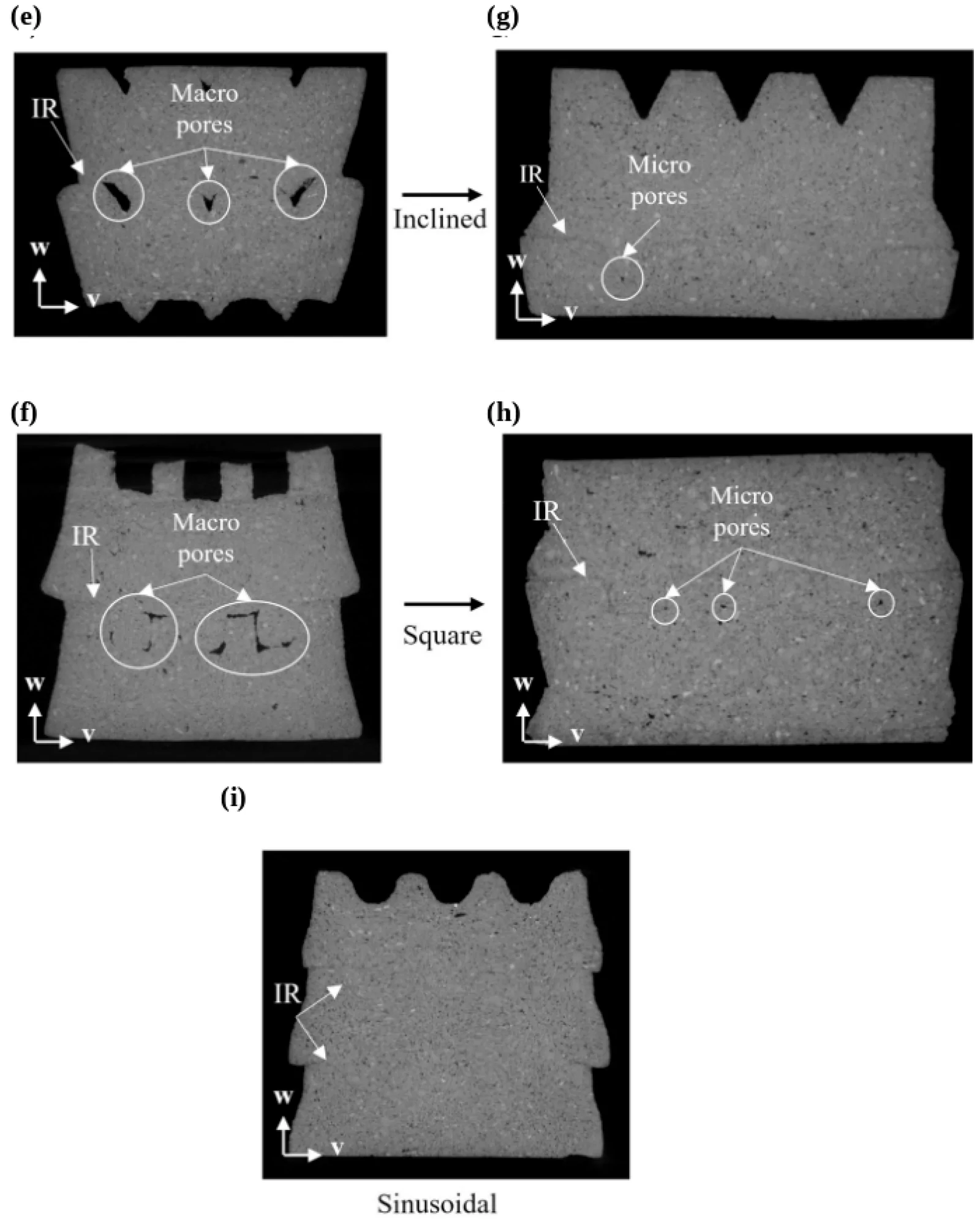
Grooved-filament concept for improving mechanical interlock between printed concrete layers. (**a**–**d**) Schematic illustration of the flat, sinusoidal, square, and inclined nozzle geometries used to create interlayer interlocking. (**e**–**i**) Cross-sectional CT scans illustrating examples of unsuccessful interlocking for the inclined (**e**) and square (**f**) patterns, and successful interlocking for the inclined (**g**), square (**h**), and sinusoidal (**i**) patterns; arrows indicate the interlayer region (IR), adapted from [71] licensed under CC BY 4.0 (https://creativecommons.org/licenses/by/4.0/).

Grooved or textured nozzles represent one of the most direct ways to improve mechanical interaction between layers (Figure 5). By modifying the surface geometry of the deposited filament, the nozzle can increase roughness and create local keying between successive layers. This mechanism is analogous to mechanical interlock in masonry, where unit geometry, surface roughness and mortar penetration contribute to shear transfer. In 3DPC, however, the interlock is created during extrusion rather than during placement of a separate mortar joint. The effectiveness of grooving therefore depends on whether the upper layer can fill the grooves before the lower layer loses excessive moisture or stiffness. If the surface has already dried or the lower layer has undergone excessive thixotropic rebuilding, geometric roughness alone may not provide sufficient bonding.

**Figure 6:**
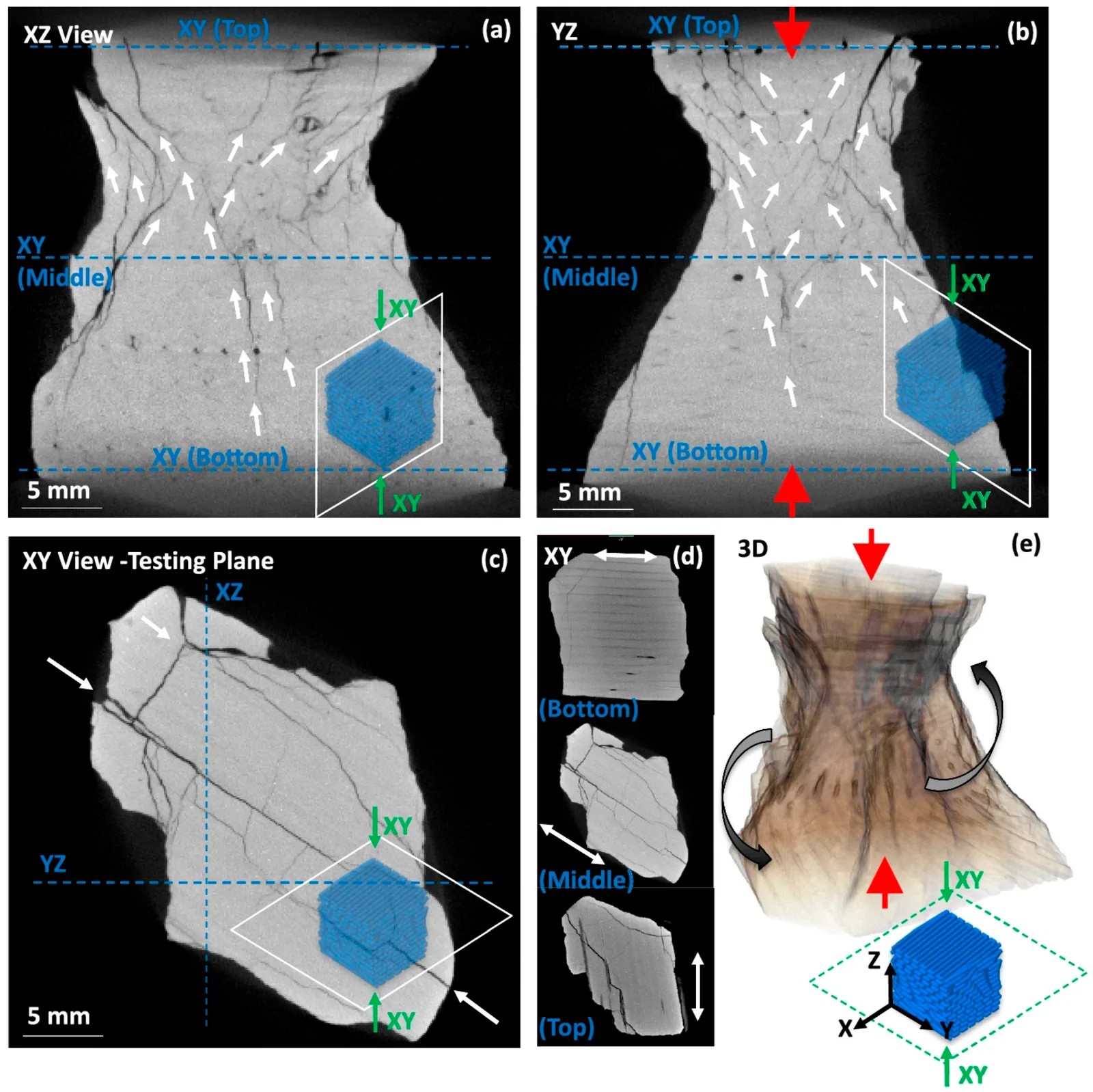
Post-mortem X-ray micro-CT images of the microstructure of a 3D-printed hardened cement paste specimen with Bouligand architecture tested in the XY plane collected during a 0.4X scan: (**a**) 2D view on the XZ plane, (**b**) 2D view on the YZ plane, (**c**) 2D view on the XY plane, (**d**) 2D view on the XY plane at three different heights of the specimen, and (**e**) three-dimensional rendition of the entire volume of the specimen, adapted from [72] licensed under CC BY 4.0 (https://creativecommons.org/licenses/by/4.0/). Red arrows indicate the loading direction; green arrows indicate the XY-plane direction; white arrows indicate crack paths; the blue plane marks the XY testing plane; grey curved arrows indicate the 3D rotation direction.

A second strategy is to alter the print path so that the crack is not allowed to remain aligned with a single interlayer plane. Rotated, Bouligand-type or helicoidal arrangements are inspired by natural layered composites in which gradual changes in fibre or platelet orientation deflect cracks and increase energy dissipation. In printed cementitious materials, the same concept can be used by changing the filament orientation between layers (Figure 6). When the crack reaches an interface with a different orientation, it may deviate from its initial path, consume additional fracture energy and activate bridging between adjacent filaments. This mechanism is especially relevant for flexure and tension, where failure is strongly controlled by crack trajectory.

**Figure 7:**
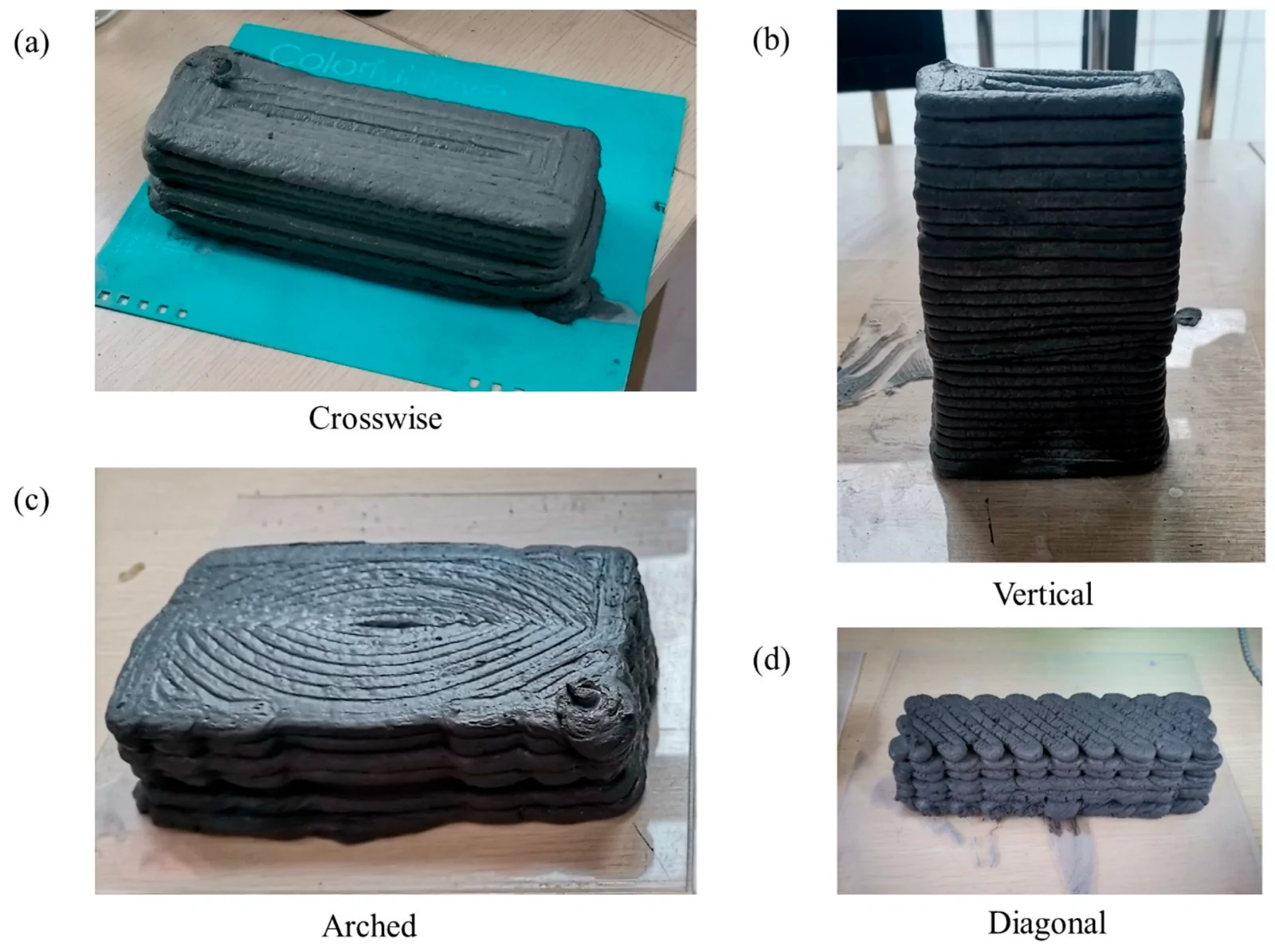
Path-dependent deposition geometries for increasing fracture-path complexity and interlayer resistance: (**a**) crosswise; (**b**) vertical; (**c**) arched; (**d**) diagonal deposition, adapted from [76].

Tilted, knitted or interwoven deposition paths follow a related principle. Instead of producing a stack of parallel filaments, these strategies create local continuity between layers by forcing filaments to cross, incline or interlock (Figure 7). Reported improvements in fracture or interlayer resistance can be substantial under selected test configurations, including values exceeding 100% in some tilted or knitted arrangements [73,74,75]. Such results should not be generalised without considering specimen geometry, loading mode, reference configuration, filament size and curing condition. Nevertheless, the mechanism is credible: a crack consumes more energy when it must change direction, cross inclined filaments or overcome local bridging rather than propagate along a smooth planar interface.

These strategies show both the strength and the limitation of the masonry analogy. Masonry improves load transfer through unit geometry, bond pattern, mortar confinement, surface roughness and reinforcement. 3DPC can go further by changing the topology of the deposited material at almost every layer. This creates a design opportunity that has no exact counterpart in conventional masonry: the interface can be engineered by toolpath, nozzle geometry and layer sequence. However, it also creates a standardisation problem. A mechanical test developed for a straight-layer wall may not represent a wall printed with tilted, corrugated, cellular, knitted or Bouligand-type deposition. The reported strength of a specimen is therefore meaningful only if the print path and layer geometry are documented together with the mixture design and loading direction.

Print-path engineering also interacts with reinforcement. Fibres aligned by extrusion may be effective along the filament but less effective across a planar interlayer. When the print path is rotated, tilted or interwoven, the relationship between fibre orientation and crack plane changes. This can improve crack bridging if fibres intersect the fracture path, but it can also create local zones of fibre congestion, poor extrusion quality or variable compaction. Therefore, the structural benefit of a complex print path should be evaluated together with fibre type, aspect ratio, dosage, nozzle shape and extrusion pressure. A geometry that improves crack deflection in an unreinforced mixture may not produce the same improvement in a fibre-reinforced mixture.

The practical implication is that print-path engineering should be treated as a structural design variable, not merely as a manufacturing detail. For structural qualification, the following information should accompany any reported mechanical result: filament width and height, nozzle geometry, layer sequence, print-path orientation, interlayer time interval, surface condition, location of specimen extraction and loading direction. Without these details, apparent improvements in flexural strength, tensile strength or fracture energy cannot be compared reliably across studies. A robust standardisation framework for 3DPC should therefore classify mechanical results according to both material composition and print architecture.

## 7. Flexural and Shear Behaviour

Flexural behaviour is one of the most sensitive indicators of interface quality in layered structural systems. In masonry, flexural resistance is governed by unit strength, mortar bond, joint orientation, bed-joint normal stress and the continuity of the bond pattern [77,78,79]. A wall bending parallel to the bed joints and a wall bending perpendicular to the bed joints do not activate the same crack path. In 3D printed concrete (3DPC), the same principle applies, although the interface is not a mortar joint (the underlying mechanism is described in Section 5). Flexural failure is strongly affected by the quality of contact between printed layers, local porosity and the direction of tensile stress relative to the print path [80,81,82,83,84]. For this reason, flexural testing often reveals weaknesses that may remain less visible in compressive tests.

The interlayer region controls flexural response because bending creates tensile stresses that are highly sensitive to weak planes. If the tensile zone intersects a poorly bonded interface, the crack may propagate along the layer boundary with limited resistance. If the crack is forced to cross well-bonded filaments, the specimen can mobilise a larger portion of the cementitious matrix and any fibre-bridging mechanism present in the mixture. This explains why two specimens produced from the same mixture may show different flexural strengths when extracted or loaded in different directions. The age of the lower layer at the moment of deposition is therefore as important as the age of the specimen at testing. This is because it governs surface moisture, thixotropic rebuilding and early hydration across the eventual fracture plane.

Masonry provides a useful reference for interpreting this behaviour. In masonry, flexural strength is not a pure material property of the brick or block; it is the result of unit–mortar interaction, joint orientation and the normal stress acting across the joint. Similarly, in 3DPC, flexural strength should not be presented only as a property of the printed mixture. It should be reported together with layer height, filament width, print direction, interlayer time interval, specimen extraction direction and loading orientation. Without this information, apparent differences in flexural strength between studies may reflect differences in interface configuration rather than true differences in material quality.

Shear behaviour follows the same interface-controlled logic. Masonry shear resistance is commonly governed by mortar cohesion, friction, normal stress across the bed joint, unit surface roughness, reinforcement and confinement [85,86,87]. Sliding along a bed joint, diagonal cracking through units and mixed unit–joint failure represent different mechanisms and cannot be reduced to a single shear-strength value. In 3DPC, shear resistance depends on interlayer bond, filament geometry, surface roughness, mechanical interlock, fibre orientation and reinforcement continuity [88,89,90]. If the potential shear plane coincides with a weak interlayer region, the response may be governed by cohesion and friction across the printed interface. If the shear plane cuts through filaments or crosses mechanically interlocked layers, the resistance may be higher because crack propagation and sliding require additional energy.

The role of fibre-reinforced or ECC-type printable mixtures must be interpreted with similar caution. High tensile ductility, strain-hardening behaviour or improved crack control measured in a material coupon does not automatically translate into high wall-level shear capacity. The fibre-bridging mechanism is effective only when fibres cross the active crack or shear plane with sufficient anchorage. Since extrusion may align fibres along the print direction, the improvement may be more pronounced along the filament than across the interlayer. Therefore, fibre type, aspect ratio, dosage, surface modification and orientation should be considered together with print path and expected failure mode.

Flexural and shear testing of 3DPC should therefore be performed and reported in direction-dependent form. At minimum, test programmes should distinguish specimens loaded along the print direction, specimens loaded perpendicular to the print direction within the layer plane and specimens loaded across the build direction. Where reinforcement is included, tests should also distinguish reinforcement that is continuous across interfaces from reinforcement inserted after printing, placed in ducts or combined with post-cast grout. These reinforcement strategies create different bond conditions and different load-transfer mechanisms. Their performance cannot be inferred from the printed matrix alone.

The practical implication is that flexural and shear properties should be linked to the structural role of the printed element. For non-load-bearing printed components, a material-level flexural index may be sufficient for quality control. For load-bearing walls, panels, beams or seismic elements, however, coupon tests should be connected to interlayer bond tests, shear-friction response, reinforcement anchorage and wall-scale validation. This hierarchy is consistent with the broader direction of current interlaboratory and standardisation efforts, which emphasise that specimen geometry, extraction location, number of interlayers, curing regime and loading configuration must be defined before mechanical data from different studies can be compared reliably [23,24,25].

## 8. Dynamic, Cyclic and Seismic Response

Dynamic and seismic performance represents one of the least mature but most important areas for structural 3DPC. Masonry has a long history of seismic research, since earthquakes activate the same weaknesses that make masonry heterogeneous. These include bed-joint sliding, diagonal cracking, rocking, out-of-plane bending, toe crushing, frame-infill interaction and failure of connections or confining elements [91,92,93,94,95,96,97,98]. These mechanisms are not governed by unit compressive strength alone. They depend on assemblage geometry, joint quality, axial load level, boundary conditions, reinforcement detailing and energy dissipation capacity. This lesson is directly relevant to 3DPC, where high compressive strength of the printed material cannot be used as proof of adequate seismic performance.

The available evidence on 3DPC under dynamic, cyclic and seismic loading is still limited compared with conventional concrete and masonry. Existing studies indicate that anisotropy affects dynamic compressive response, crack orientation, stiffness degradation and wave-propagation measurements [99,100]. Under cyclic or quasi-static lateral loading, the response of printed walls is governed by wall configuration, aspect ratio, axial load, reinforcement strategy, interlayer bond and connection detailing [101,102,103,104]. These variables determine whether damage is distributed through the printed element or localised along layer boundaries, dry joints, corners, restart regions or reinforcement discontinuities.

The interlayer region is particularly important under cyclic loading because repeated stress reversal can progressively degrade weak interfaces. A monotonic test may show adequate capacity if the interface remains compressed or if the crack path does not fully activate the weak plane. During cyclic loading, however, opening, closing, sliding and frictional degradation can accumulate at the same interface. If the printed layers are not sufficiently integrated, stiffness degradation and residual deformation may develop rapidly. This behaviour is analogous to masonry bed-joint sliding and diagonal cracking. However, the physical origin differs; the printed interface is governed by deposition history and moisture-related effects rather than mortar placement alone (the underlying mechanism is described in Section 5).

**Figure 8:**
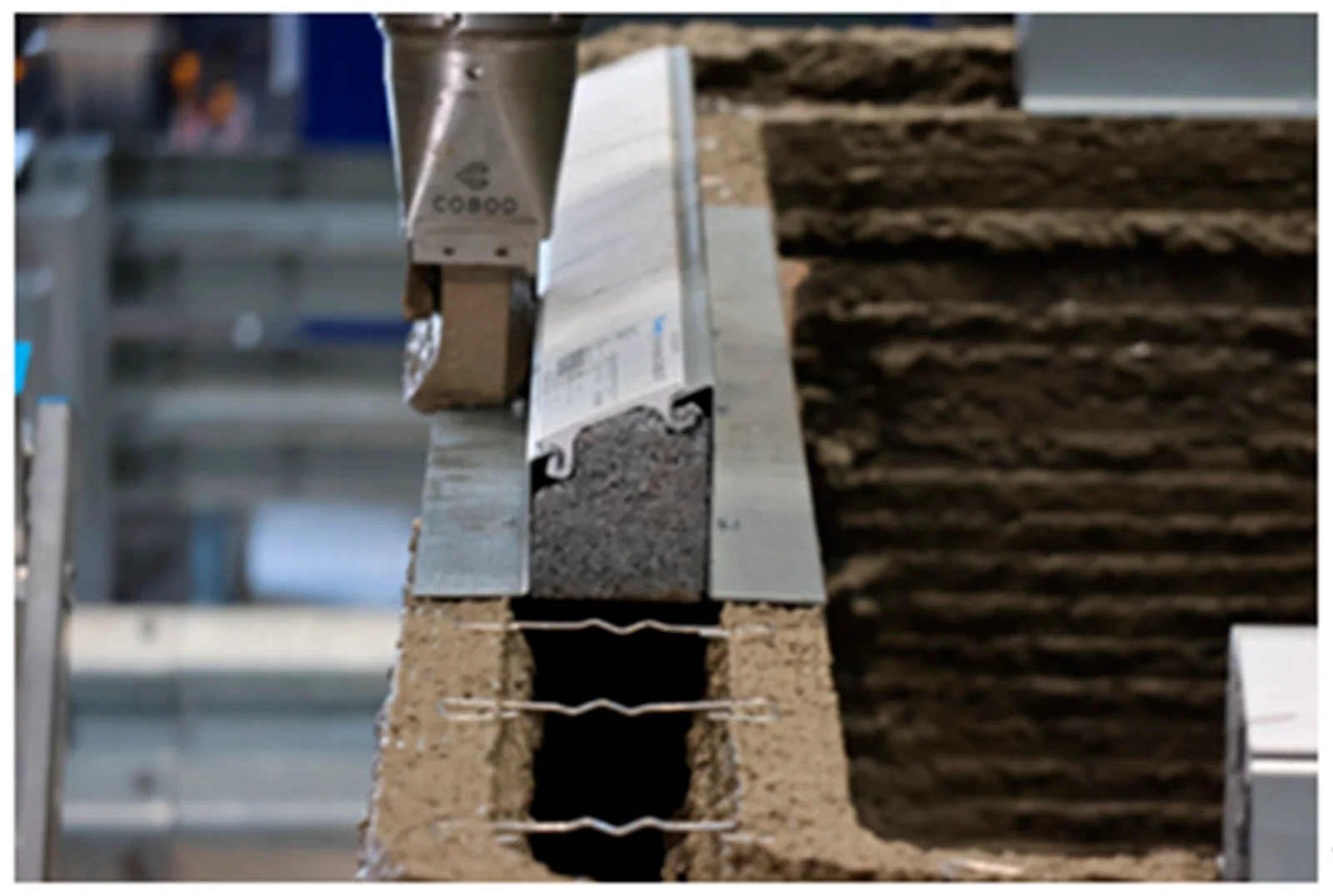
Prefabricated steel elements used with a 3D-printed concrete wall system in a seismic-loading investigation, adapted from [105].

**Figure 9:**
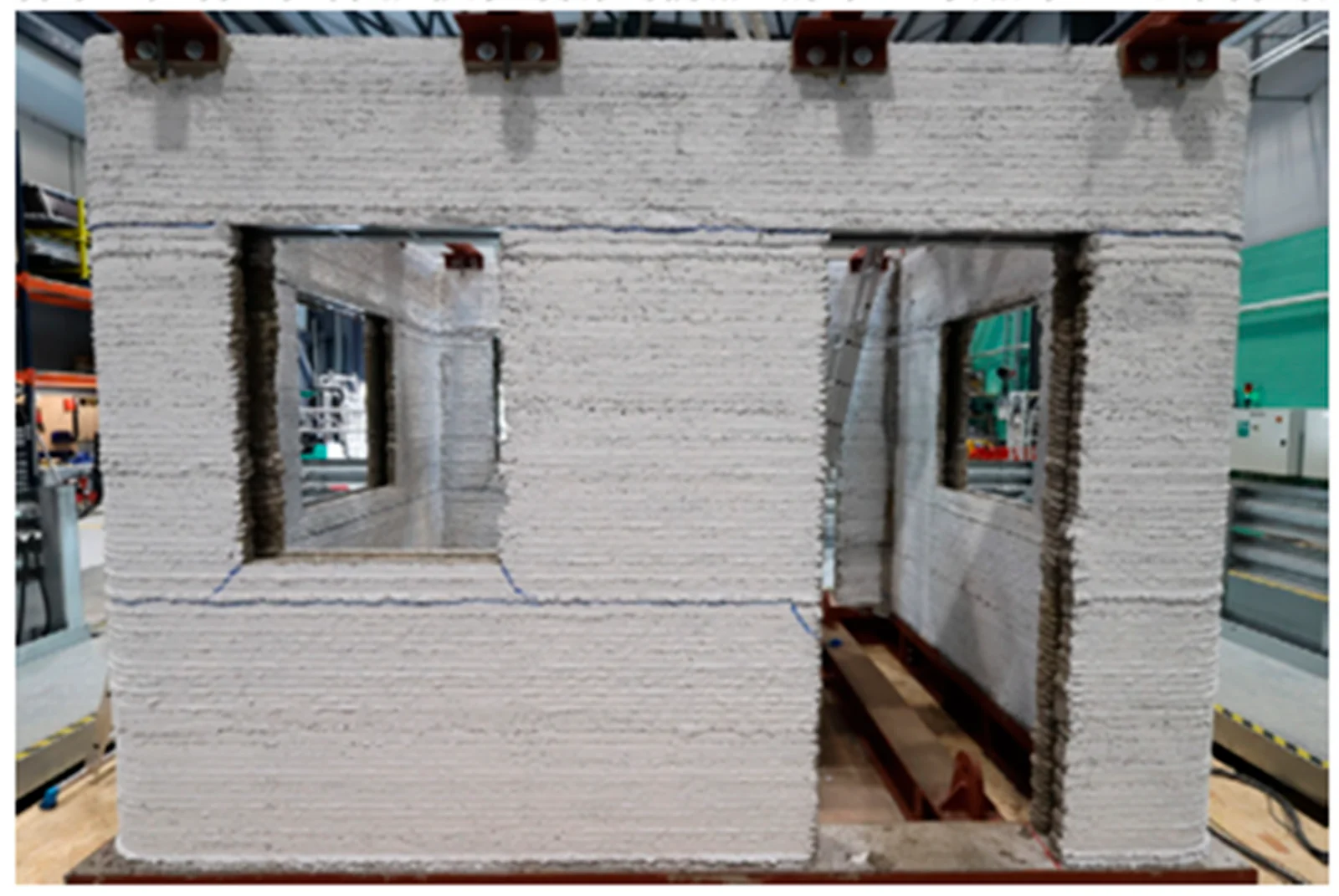
Failure mode of a 3DPC building after seismic loading, showing the importance of interlayer continuity and connection detailing, adapted from [105].

Reinforcement improves cyclic and seismic performance only when it provides a continuous and reliable load path (Figure 8 and Figure 9). In 3DPC, reinforcement may be inserted manually, placed between layers, post-installed through printed ducts, combined with prefabricated steel elements or integrated by robotic systems. Each approach creates different bond and anchorage conditions. Reinforcement that is mechanically present in the wall cannot be assumed effective if tensile forces, shear forces and cyclic stresses cannot be transferred across printed interfaces. The connection between printed concrete, reinforcement, grout, steel inserts and boundary elements should therefore be treated as part of the seismic load path rather than as a secondary construction detail.

The masonry analogy is useful in this context because masonry seismic design does not rely on unit tests alone. It uses assemblage tests, wall tests, reinforcement detailing, confinement, anchorage rules and empirical or semi-empirical checks for in-plane and out-of-plane actions. A similar philosophy is needed for 3DPC. Compressive, tensile or flexural coupons can provide necessary input values, but they cannot replace wall-level cyclic tests until validated relationships exist between coupon properties, interlayer fracture energy, reinforcement anchorage and full-scale response. This is especially important for printed elements with corners, openings, variable print paths, construction pauses, embedded reinforcement or hybrid steel-concrete detailing.

For seismic qualification, 3DPC test programmes should therefore move beyond isolated material characterisation. Relevant specimens should include wall panels, boundary conditions, reinforcement layouts, interlayer configurations, openings, joints and connections that represent actual construction. Test reporting should include print path, layer height, number of layers, interlayer time interval, reinforcement strategy, connection details, axial load, loading protocol and observed failure mode. Without these parameters, cyclic data cannot be generalised from one printed geometry to another.

At the present stage, the safest conclusion is that 3DPC has structural potential under dynamic and seismic loading, but its performance cannot be assumed from high matrix strength or successful printability. The decisive issue is whether the printed system can provide continuous load transfer, stable energy dissipation and controlled damage under repeated loading. Establishing this requires a hierarchy of tests from material coupons to interlayer bond specimens, reinforced wall panels and full-scale or near-full-scale cyclic validation. Until such evidence becomes broader, seismic design of 3DPC should remain conservative and should explicitly account for anisotropy, interlayer continuity and connection detailing.

## 9. Standardisation: Transferable Principles and Required Modifications

Standardisation is one of the decisive barriers between successful printing at laboratory scale and reliable structural application of 3D-printed concrete (3DPC). Existing standards for mortar, masonry and concrete provide useful reference methods, but none of these groups alone can fully describe the layered, process-dependent and anisotropic nature of printed cementitious materials. The main issue is not whether current standards are irrelevant; rather, it is where their assumptions remain valid and where they must be modified before being applied to 3DPC.

Fresh mortar and paste standards are useful for measuring workability, consistency, flow and hardened mortar strength. However, they do not directly quantify pumpability, extrudability, shape stability, open time, structural build-up or the loss of bonding capacity between layers. A mortar may satisfy conventional flow or strength requirements and still be unsuitable for printing if it tears during extrusion, collapses after deposition or loses interlayer bonding because of rapid surface drying. Therefore, fresh-state standards can provide baseline indices, but they cannot be used as acceptance criteria for 3DPC without additional printability-related measurements.

Masonry standards are more relevant to the structural philosophy of 3DPC because they recognise the difference between material units and assemblages. Masonry prism, wallet, flexural, bond and shear tests evaluate the interaction between units and joints rather than the brick or block alone. This logic is valuable for 3DPC, where the printed wall cannot be represented by a cast or printed cube alone. However, masonry standards assume manufactured units joined by mortar, whereas 3DPC contains process-created interlayers whose properties depend on rheology, deposition pressure, surface moisture, interlayer time interval and curing history. For this reason, masonry standards can guide the development of 3DPC assemblage tests, but they cannot be transferred directly.

Concrete standards remain necessary for basic compressive, flexural and tensile characterisation. Nevertheless, conventional concrete testing generally assumes that the specimen is comparatively homogeneous at the scale of the test. In 3DPC, the specimen may contain layer boundaries, aligned pores, different compaction histories, restarts, corners and direction-dependent weak planes. A compressive or flexural result is therefore incomplete unless the print direction, extraction direction, number of layers, interlayer interval, curing regime and loading orientation are reported. Without these details, the same nominal test can represent different failure mechanisms.

Table 6 summarises the role of selected mortar, masonry and concrete-related standards in relation to 3DPC. The purpose of the table is not to suggest direct adoption, but to identify which parts of existing testing logic can be transferred and which parts require modification.

Read together, the standards summarised in Table 6 show a consistent pattern rather than a set of unrelated gaps. Masonry standards (EN 1052-1, -2, -3, -5; ASTM C1314; ASTM E519/E519M) [116,117,118,119,120,121] already provide assemblage-level testing logic for compression, flexure, shear and bond, but none of them anticipates a process-generated, time-dependent interface. Conventional concrete standards, conversely, provide direction-independent material indices but do not require reporting of print-related parameters. The dedicated 3DPC activities (ACI Committee 564, the NIST measurement-science programme, RILEM TC 304-ADC) [21,22,23,24,25] explicitly recognise this gap but have not yet converged on a single reporting protocol. The agreement across all three streams is therefore not about test geometry, but about the need to report interlayer condition, print-path geometry and specimen orientation alongside any mechanical result—the limitation is the absence of a standard that combines assemblage-level logic with process-parameter reporting.

**Figure 10:**
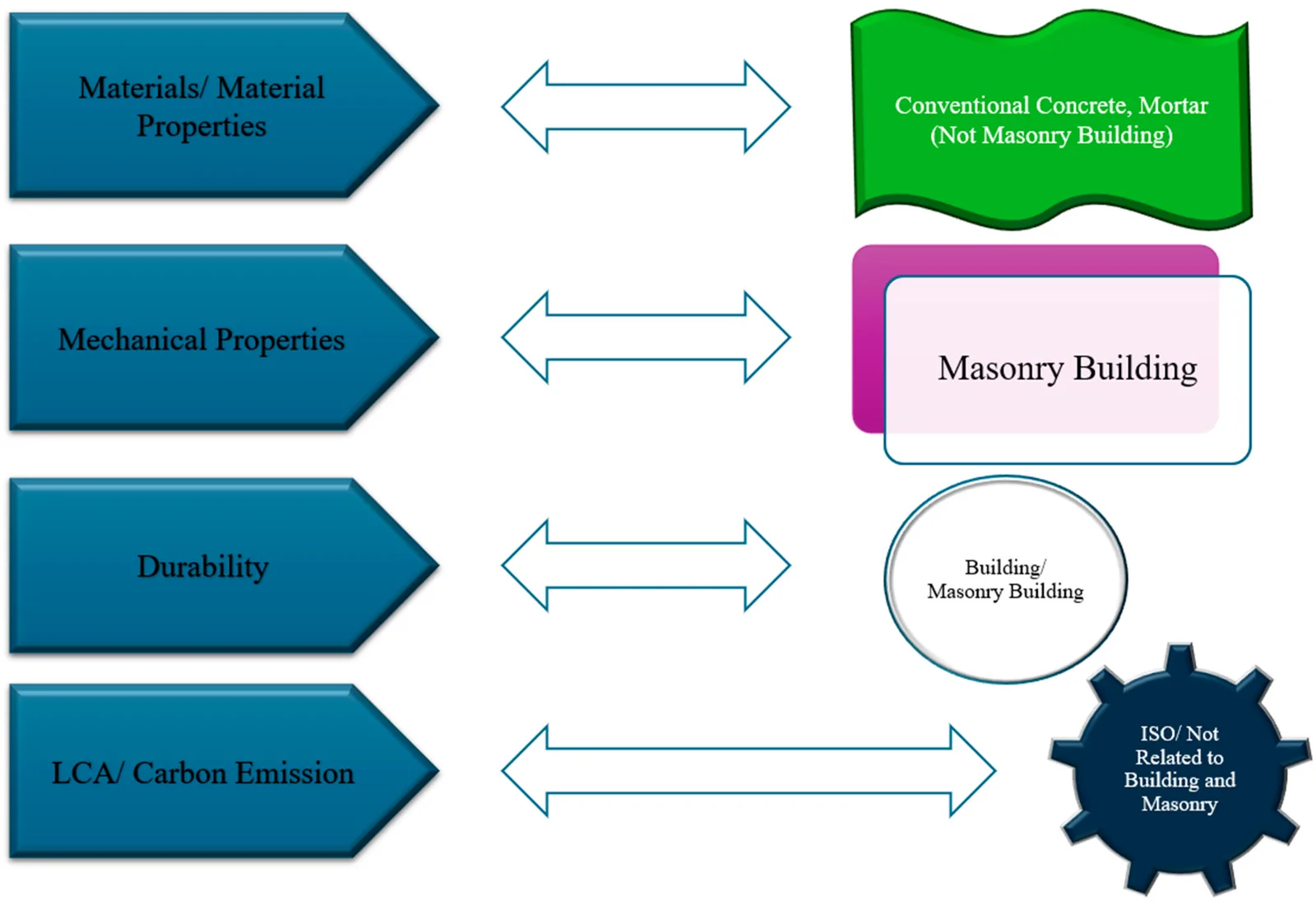
Standardisation route for transferring mortar, masonry and concrete testing principles to 3DPC-specific structural qualification.

Figure 10 presents the standardisation route proposed in this review. Existing standards are treated as a transfer map rather than as a ready-made framework. Mortar and concrete tests provide material-level indices, masonry tests provide assemblage-level logic, and 3DPC-specific requirements must connect these two levels through print-process variables.

The most defensible standardisation route for 3DPC is a tiered qualification framework. The first tier should characterise the fresh material in terms of pumpability, extrudability, open time, structural build-up, shape stability and surface moisture retention. These parameters determine whether stable and bondable layers can be produced. The second tier should evaluate hardened coupons in multiple orientations, including specimens loaded along the print direction, perpendicular to the print direction and across the build direction. This tier provides basic direction-dependent material indices.

The third tier should focus on interface-dominated specimens. Direct tension, splitting, shear and fracture tests should be performed across printed layers to determine whether the interlayer region behaves as a continuous material zone or as a weak plane. These tests should report interlayer time interval, layer height, nozzle geometry, surface condition, extraction location, number of interfaces and curing regime. The fourth tier should test printed panels or wall segments under axial, flexural, shear and cyclic loading. These specimens should include realistic print paths, corners, openings, restarts and reinforcement details when these features are present in the intended structure.

The fifth tier should connect test results to design values. Until robust relationships are established between coupon properties, interlayer bond, reinforcement anchorage and wall-scale response, design properties should be assigned directionally or reduced conservatively. This is especially important for tensile, flexural, shear and seismic resistance, where failure may be governed by interlayer defects rather than matrix strength. A single compressive-strength value should not be used as the main basis for structural qualification.

Table 7 summarises the proposed transfer matrix from masonry testing logic to 3DPC structural qualification. The matrix highlights that the valuable lesson from masonry is not a specific test geometry, but the principle that structural behaviour must be evaluated at the assemblage level.

Unlike ACI Committee 564, which currently provides terminology and general guidance without a tiered testing sequence, and RILEM TC 304-ADC, which focuses on establishing interlaboratory comparability of individual test results, the framework proposed here explicitly links fresh-state characterisation, direction-dependent coupon testing, interface-dominated testing and wall-scale validation into a single qualification path modelled on the assemblage-level logic already codified in masonry testing standards (EN 1052 series, ASTM C1314, ASTM E519/E519M) [116,117,118,119,120,121]. Its practical contribution is therefore not a new test method, but a reporting and sequencing logic: it specifies which structural question each tier answers and which print-process parameters must accompany the result. This could feed into future standardisation efforts in two concrete ways: as a candidate structure for a dedicated 3DPC testing standard (in the spirit of ACI Committee 564’s ongoing work) and as a checklist against which RILEM interlaboratory studies could report results, so that data generated for different purposes remain comparable [21,23,24,25].

A standardisation framework for 3DPC should therefore require more than mixture proportions and nominal strength. At minimum, structural test reports should include binder system, aggregate grading, admixture dosage, rheological state, nozzle geometry, layer height, filament width, printing speed, extrusion rate, interlayer interval, surface condition, curing regime, specimen extraction direction, loading direction, reinforcement strategy and failure mode. These data are necessary because the printed element is manufactured and structurally configured at the same time.

The main conclusion is that existing mortar, masonry and concrete standards are not obsolete for 3DPC, but their use must be conditional. Mortar and concrete tests are appropriate for baseline material characterisation. Masonry tests are valuable for their assemblage-level philosophy. RILEM, ACI and NIST activities show that the field is moving toward dedicated procedures. The remaining task is to connect these strands into a qualification framework that treats 3DPC as a layered structural material whose properties depend on both composition and print process.

## 10. Conclusions

This review examined 3D-printed concrete (3DPC) through the lens of masonry construction to clarify how interface-controlled structural behaviour should be interpreted and standardised. The comparison does not imply that 3DPC is a form of masonry or that masonry provisions can be transferred directly to printed cementitious materials. Its value lies in the common structural lesson: neither system can be evaluated reliably from isolated material strength alone. In masonry, the governing scale is the unit–mortar assemblage; in 3DPC, it is the printed filament-interlayer assemblage.

The main conclusion is that 3DPC should be treated as a layered structural material whose properties are created jointly by mixture design and printing history. Binder type, aggregate grading, admixture chemistry, fibre content and mineral additions affect structural performance through their interaction with pumpability, extrudability, structural build-up, surface moisture, interlayer interval, nozzle geometry and print path. High compressive strength is therefore not sufficient for structural qualification if weak interlayers, poor reinforcement continuity or direction-dependent failure paths are present.

The interlayer region is the critical feature that links material design to structural performance. Incomplete contact, moisture loss, thixotropic rebuilding, early hydration, pore alignment and variable compaction produce its weakness during deposition. These mechanisms control crack initiation and propagation under flexure, tension and shear, and they may govern cyclic degradation under repeated loading. Consequently, interlayer bond should be treated as a primary structural property rather than as a secondary print-quality issue.

Existing mortar, concrete and masonry standards remain useful, but only as partial references. Mortar and concrete tests provide baseline material indices, while masonry tests offer an assemblage-level philosophy. However, none of these standards alone captures process-created interlayers, direction-dependent properties, print-path sensitivity or reinforcement discontinuity in 3DPC. A defensible qualification route should therefore be tiered, progressing from fresh-state characterisation to direction-dependent coupon testing, interface-dominated tests, reinforced wall or panel tests and design values calibrated against structural-scale behaviour.

The main contribution of this review is therefore not another general summary of 3DPC materials or printability requirements, but a structural interpretation of 3DPC as an interface-controlled assemblage. By using masonry as a reference only for joint-controlled load transfer and testing hierarchy, the review clarifies why printed concrete requires qualification procedures that report interlayer condition, print-path geometry, specimen orientation and wall-scale response together.

For future structural use, 3DPC test reports should include mixture composition and nominal strength together with rheological state, nozzle geometry, layer height, filament width, printing speed, extrusion rate, interlayer interval, surface condition, curing regime, specimen extraction direction, loading direction, reinforcement strategy and failure mode. Without these parameters, results from different studies cannot be compared reliably or converted safely into design values. The broader implication is that 3DPC standardisation should begin from the recognition that the printing process creates the material architecture. Only by treating printed interlayers, print-path geometry and wall-scale response as structural variables can 3DPC move from printable material development toward reliable structural application.

Beyond these general recommendations, three research gaps stand out as priorities for future work. First, mechanical and durability data for 3DPC remain difficult to compare across studies because process parameters—nozzle geometry, interlayer interval, curing regime—are inconsistently reported; establishing a shared, minimum reporting protocol, building on the tiered qualification framework proposed in Section 9, would be a direct and achievable next step. Second, the transfer of results from coupon-scale tests to wall- and panel-scale structural response remains largely undeveloped; scale-transition approaches analogous to those used in masonry design are needed to connect interlayer bond and direction-dependent coupon data to structural-scale predictions. Third, time-dependent structural performance—particularly creep, shrinkage-induced cracking and interlayer fatigue under repeated loading—remains substantially understudied compared to short-term mechanical response, and dedicated experimental programmes targeting these mechanisms specifically at the interlayer scale are needed before 3DPC can be reliably qualified for long-term structural service. Addressing these three gaps would move 3DPC from demonstration-scale printability toward code-recognised structural qualification.

## Figures and Tables

**Table 1:** Selected studies and standardisation activities related to 3DPC printability, interface behaviour, anisotropy and structural qualification.

Reference	Main Focus	Examined Parameters and Reporting Details	Main Findings Relevant to This Review	Relevance to Masonry–3DPC Comparison
Le et al. [1,2]	Early mix design and hardened properties of high-performance printing concrete	Filament architecture, interfacial regions, three-point bending, ball-on-three-ball testing and micro-CT Sample size: At least 2 independent results per reported data point Printer type: Bespoke system combining an Ultimaker 2 Extended+ and Structur3d Discov3ry Paste Extruder Interlayer interval: NR Specimen orientation: Printed prisms: 0°, 45° and 90° filament orientations; architecture-dependent disk orientations Test standard: Three-point bending and ball-on-three-ball tests; ASTM C150 and ASTM C494 for constituent materials Statistical dispersion: Average values and 95% confidence-level comparisons reported	Demonstrated that printable cementitious mixtures require different fresh-state and hardened-state considerations than cast concrete.	Establishes the starting point for treating 3DPC as a process-dependent material rather than ordinary concrete placed by another method.
Buswell et al. [3]	Research roadmap for concrete extrusion in digital construction	Construction-scale additive manufacturing processes, opportunities and preliminary applications Sample size: NA Printer type: NA Interlayer interval: NA Specimen orientation: NA Test standard: NA Statistical dispersion: NA	Identified the need to connect material behaviour, process parameters and structural performance for construction-scale adoption.	Supports the argument that 3DPC requires a structural framework beyond material printability.
Roussel [4]	Rheological requirements for printable concrete	Yield stress, viscosity, elastic modulus, critical strain and structuration Sample size: NA Printer type: NA Interlayer interval: NA Specimen orientation: NA Test standard: NA Statistical dispersion: NA	Explained that printable concrete must satisfy conflicting rheological requirements before and after extrusion.	Provides the fresh-state mechanism behind the formation of stable or weak printed layers.
Wolfs, Bos and Salet [5]	Early-age mechanical behaviour and buildability of printed concrete	Unconfined compression, direct shear, age-dependent strength/stiffness and printing validation Sample size: 30 unconfined-compression specimens; 75 direct-shear specimens Printer type: 3DCP setup: M-Tec Duomix 2000 mixer-pump, linear displacement pump and 1 in. hose Interlayer interval: NR as a deposited-layer interval; material age tested from 0 to 90 min Specimen orientation: NR as printed-specimen extraction orientation Test standard: ASTM D2166-informed unconfined-compression geometry; study-specific direct-shear procedure Statistical dispersion: Mean, standard deviation and relative standard deviation reported	Showed that early-age behaviour governs the ability of lower layers to support subsequent layers.	Relates 3DPC layer stability to structural capacity during construction, an issue not captured by ordinary concrete tests.
Moini et al. [7]	Microstructural features of architectured cement-based materials	Filament architecture, interfacial regions, three-point bending, ball-on-three-ball testing and micro-CT Sample size: At least 2 independent results per reported data point Printer type: Bespoke system combining an Ultimaker 2 Extended+ and Structur3d Discov3ry Paste Extruder Interlayer interval: NR Specimen orientation: Printed prisms: 0°, 45° and 90° filament orientations; architecture-dependent disk orientations Test standard: Three-point bending and ball-on-three-ball tests; ASTM C150 and ASTM C494 for constituent materials Statistical dispersion: Average values and 95% confidence-level comparisons reported	Showed that printing can create patterned pore networks and interfacial regions that differ from cast specimens.	Provides microstructural evidence that printed interfaces are process-generated weak regions.
Şahin and Mardani-Aghabaglou [8]	Materials, design parameters and properties of 3DPC mixtures	Binders, SCMs, admixtures, fibres, recycled materials, mixture design, rheology and sustainability Sample size: NA Printer type: NA Interlayer interval: NA Specimen orientation: NA Test standard: NA Statistical dispersion: NA	Reported that existing standards do not sufficiently guide 3DPC mix design and acceptance criteria.	Establishes the need for 3DPC-specific evaluation rather than direct adoption of conventional concrete provisions.
Şahin and Mardani [9]	Mechanical properties, durability performance and interlayer adhesion of 3DPC	Loading direction, printing interval, speed, nozzle parameters, shrinkage, durability and adhesion Sample size: NA Printer type: NA Interlayer interval: NA Specimen orientation: NA Test standard: NA Statistical dispersion: NA	Showed that interlayer adhesion and mechanical performance are strongly governed by printing parameters and loading direction.	Directly supports the treatment of 3DPC as a layered structural material with interface-controlled behaviour.
Şahin and Mardani [10]	Relationship between rheological behaviour and interlayer-bonding strength	Static yield stress, structural build-up, surface condition/moisture, interval, speed and nozzle parameters Sample size: NA Printer type: NA Interlayer interval: NA Specimen orientation: NA Test standard: NA Statistical dispersion: NA	Explained that interlayer bond depends on the balance between structural build-up and the bonding ability of the layer surface.	Clarifies why buildability and bond strength may conflict in 3DPC, unlike the more deliberately formed mortar joints in masonry.
Biricik and Mardani [11]	Thixotropic behaviour of SCC and 3DPC	Cement, SCMs, admixtures, aggregate, w/b ratio, mixing and rheological methods Sample size: NA Printer type: NA Interlayer interval: NA Specimen orientation: NA Test standard: NA Statistical dispersion: NA	Reported that thixotropy is beneficial only within an optimum range and that excessive structural build-up may promote cold joints.	Provides the mechanism by which time-dependent stiffening can create weak interfaces between printed layers.
Şahin, Mardani and Mardani [12]	Optimum mix proportion of high-volume fly ash 3DPC	w/b, sand/binder, fly ash, aggregate size, admixture dosage, rheology, printability and 28-day strength Sample size: 32 mixtures Printer type: Mortar injection gun; 42 mm nozzle width Interlayer interval: NR Specimen orientation: NR Test standard: EN 1097-6 for aggregate properties; mechanical-test standard NR Statistical dispersion: NR	Proposed mix-design ranges satisfying extrudability, buildability and shape stability requirements.	Shows that mixture design must be linked to layer stability and print quality, not only to compressive strength.
Şahin, Mardani and Beytekin [13]	Silica fume in fibre-reinforced 3DPC	Silica-fume dosage, rheology, structural build-up, strength and drying shrinkage Sample size: 5 mixtures; 3 samples prepared for each experiment Printer type: Injection-gun extrusion; nozzle outlet 42 mm wide and 15 mm high Interlayer interval: NR Specimen orientation: NR Test standard: Material-characterisation standards reported; mechanical-test standard NR Statistical dispersion: NR	Showed that silica fume can modify build-up and rheology, but may also influence flexural and shrinkage behaviour depending on dosage.	Demonstrates that improving one printability-related property may create penalties in another structural property.
Şahin, Akgümüş and Mardani [14]	Fibre-reinforced 3DPC with different fibre contents and aspect ratios	Rheology, thixotropy, compression, flexure, shrinkage and loading direction Sample size: 10 mixtures; 3 printed specimens from each batch Printer type: Mortar injection gun; approximate printing speed 5 mm/s Interlayer interval: NR Specimen orientation: Perpendicular and lateral to the printing direction Test standard: Modified TS EN 196-1; ASTM C596-01 Statistical dispersion: NR	Identified optimum fibre content and length for mechanical and dimensional stability, while excessive fibre use reduced performance.	Shows that reinforcement efficiency in 3DPC depends on fibre orientation, dosage and loading direction.
Şahin et al. [15]	Ozone-modified carbon fibre in 3DPC	Fibre modification, rheology, thixotropy, compression, flexure and elevated-temperature response Sample size: 7 mixtures; specimen count NR Printer type: NR Interlayer interval: NR Specimen orientation: Compression and flexure loaded perpendicular to the printing direction Test standard: TS EN 196-1 Statistical dispersion: NR	Reported that fibre surface modification improved fibre–matrix adhesion and mechanical performance.	Extends the interface problem from layer-to-layer bonding to fibre–matrix adhesion within printed materials.
Akgümüş, Şahin and Mardani [16]	Waste steel fibre and blast furnace slag in 3DPC fresh-state performance	BFS replacement, fibre length/volume, extrudability, buildability, shape stability, rheology and carbon emissions Sample size: 39 mixtures Printer type: NR; pressing/printing procedure reported; printing speed 16 mm/s Interlayer interval: NR Specimen orientation: NA—fresh-state programme Test standard: Study-specific printability and rheometer procedures Statistical dispersion: SD and CoV reported for carbon-emission results	Showed that high-volume BFS and waste fibres can reduce environmental impact while significantly changing rheological behaviour.	Links sustainability-driven binder replacement with the fresh-state parameters that control printed-layer stability.
Akgümüş et al. [17]	Waste steel fibre and blast furnace slag in 3DPC mechanical performance	BFS replacement, fibre length/volume, compression, flexure, surface moisture, shrinkage and microstructure Sample size: 28 mixtures; specimen count NR Printer type: NR Interlayer interval: NR Specimen orientation: Compression perpendicular to printing direction; flexural prisms tested by three-point bending Test standard: Modified TS EN 196-1 Statistical dispersion: NR	Reported that moderate fibre use improved strength and shrinkage performance, while excessive fibre content could reduce compressive strength.	Confirms that structural qualification must consider both fresh-state behaviour and hardened mechanical response.
RILEM TC 304-ADC interlaboratory studies [23,24,25]	Mechanical-property testing and comparability of 3DPC	Compression, elastic modulus, flexure, splitting tension, uniaxial tension, extraction and loading orientations Sample size: 34 contributions from 30 laboratories in 19 countries; approximately 5000 specimens Printer type: Laboratory-specific systems, including gantry, robotic-arm and delta configurations Interlayer interval: Laboratory-specific values; no single common interval Specimen orientation: Multiple prescribed extraction and loading orientations Test standard: Shared interlaboratory study procedures for the stated mechanical tests Statistical dispersion: Means, standard deviations, CoV and interlaboratory variability reported	Demonstrated that results from different laboratories are difficult to compare without strict reporting of printing and testing procedures.	Provides strong evidence that 3DPC standardisation must include print path, specimen orientation, interlayer condition and extraction procedure.
Bai et al. [17]	Bond behaviour of the interface between 3D-printed ECC permanent formwork and post-cast recycled concrete	Single-layer printing height, PE-fibre content, recycled-sand replacement ratio, interfacial splitting tensile strength, interfacial shear strength, DIC, X-CT pore structure and SEM micromorphology. Sample size: Nine interface configurations, including one cast control; three specimens were prepared for each mechanical test. Printer type: 3D printing system; circular nozzle with a diameter of 30 mm; nozzle travel speed of 40 mm/s. Interlayer interval: NR for successive printed layers; post-cast concrete was placed 7 days after printing. Specimen orientation: Composite specimens were tested across the printed-ECC/post-cast-concrete interface; orientation relative to the printing direction was NR. Test standard: GB/T 50081-2002 was used for the cast-concrete compressive and splitting-tensile tests; the interface splitting test followed the study procedure, and the interface shear test followed the method proposed by Bai et al. [17] Statistical dispersion: Three specimens per mechanical test and mean values were reported; the dispersion measure was not explicitly identified.	Interfacial splitting tensile and shear strengths first increased and then decreased with increasing fibre content and single-layer height, with the best performance at a 15 mm layer height and 1.5% PE fibre. Replacing natural sand with recycled sand reduced interface strength; at 100% replacement, splitting tensile and shear strengths decreased by 36.1% and 35.8%, respectively. X-CT and SEM results linked the loss of bond to increased interfacial porosity and microcracking.	Extends the interface-controlled interpretation of 3DPC from layer-to-layer bonding to the structural connection between printed permanent formwork and post-cast concrete. The study also shows that printed surface geometry and pore structure govern mechanical interlocking and composite action.

**NR** = not reported in the available full text; **NA** = not applicable to the source type.

**Table 2:** Representative masonry material modifications and their relevance to interface-controlled structural behaviour.

Reference	Material Type	Main Modification	Reported Parameters	Relevance to This Review
Hamard et al. [47]	Cob	Clay–sand mixture with straw fibre	Optimum clay content ≈20%; manufacture water content ≈20%; fibre content 1.4–1.7% by weight (straw, hay, heather, etc.); values synthesised from 133 references, 77% from France and the UK	Shows the role of fibre and soil grading in crack control and dimensional stability of earthen masonry materials.
Alassaad et al. [48]	Cob	Clay–sand mixture with straw fibre	Soil: 66.7% low-plasticity silt (ML), 33.3% silty sand with gravel (SM), fixed 2.5% flax straw fibre; 0–10% microencapsulated PCM (paraffin core): compressive strength 1.95 MPa (0%) to 1.50 MPa (10%, −23%), tested per EN 1015-11 on Ø11 × 22 cm cylinders; thermal conductivity 0.76 to 0.64 W/(m·K) (−15%); water vapour permeability reduced by up to 33% at 10% PCM	Demonstrates that fibre addition is used to improve the behaviour of unit-based earthen materials.
Quagliarini et al. [49]	Cob	Optimised clay–sand–water proportion	36% clay, 13.5% sand and about 28% water content; initial compressive strength 0.25–0.39 MPa (avg. 0.30 MPa, moist walls tested per uniaxial compression, Metrocom MI 300 press); initial shear strength 0.3–0.5 MPa at dried–dried interfaces vs. 0.07–0.20 MPa at dried–moist interfaces (Casagrande apparatus, per ASTM D3080)	Indicates that moisture content and particle proportion are central to fresh placement and hardened performance in earthen systems. The markedly lower shear strength measured at the interface between dried and freshly added moist cob (as low as 0.07 MPa) directly parallels the interlayer strength deficit central to this review’s 3DPC discussion.
Donkor and Obonyo [31]	Compressed earth block	Polypropylene fibre addition	0.2–1.0% polypropylene fibre: compressive strength 4.19 MPa (0%) to 5.15 MPa (+22.5% at 0.4%), then declining to 3.71 MPa (−11.5% at 1.0%); 3-point bending 0.68 MPa (0%) to 0.83 MPa (+22% at 0.4%); tested per uniaxial compression (Gilson machine, 623 N/s) and 3-point bending to 80% load drop, at 28 days	Provides an example of synthetic fibre reinforcement in masonry units, relevant to crack control and toughness.
Medjo Eko et al. [32]	Unfired earth brick	Steel fibre addition	1.7%, 2.0% and 2.7% steel fibre: (critical fibre length 35 mm); peak tensile strength 0.68 MPa at 2% fibre content (per CDE procedure); compressive strength up to 11.6 MPa at 10% cement content (ASTM D2166); flexural strength tested per ASTM D1635; all mechanical properties evaluated at 7, 14, 21 and 28 curing days	Shows that metallic fibres can be used to improve mechanical performance of earthen units.
Chan et al. [33]	Clay brick	Pineapple leaf fibre addition	0.25–0.75% fibre content: (pineapple leaf or oil palm fibre, with 0–15% cement); tested per BS 3921:1985 and MS76:1972 (compression, water absorption, efflorescence); all baked and non-baked specimens exceeded the 5.2 MPa minimum compressive strength for conventional bricks, with baked specimens consistently stronger; specimens tested after baking up to 800 °C or air/oven-drying (non-baked)	Demonstrates the use of plant-based fibres in fired or clay-based masonry units.
Pekrioglu Balkis [34]	Brick unit	Polypropylene fibre addition	0.5% fibre; compressive strength of 3.47 MPa and flexural strength of 1.34 MPa (per ASTM C109 compressive and ASTM C490 flexural test methods, Turkish Standard TS 2514-2515); reported at 28 days, with all fibre-added mixes meeting ASTM and Turkish standard minimums	Shows how fibre reinforcement can influence both compressive and flexural behaviour.
Gandia et al. [51]	Brick unit	Glass fibre addition	10% GFRP waste; compressive strength 1.83–2.05 MPa (+28% to +45% vs. control, depending on test method: NTE E.080 or BSPA); toughness increased +289% to +555%; no flexural strength was tested in this study; 35-day drying period, 5 replicates	Indicates that fibre type and dosage do not improve all mechanical properties uniformly.
Mostafa and Uddin [52]	Compressed earth block	Banana fibre addition	0.35% fibre (mix #7, treated 50 mm fibres): compressive strength 5.92 MPa (+78% vs. unreinforced control) and flexural strength 0.95 MPa (+94% vs. control); tested per ASTM C67-07 (compression: flexure: centre-point loading,); 5 specimens per mix (35 total per test), evaluated at 28 days	Supports the point that natural fibres can improve selected mechanical responses but require mixture-specific evaluation.

**Table 3:** Material and process variables controlling structural behaviour in 3DPC.

Variable	Primary Structural Effect	Mechanism	Required Reporting for Structural Evaluation
Binder type and replacement level	Early stiffness, strength development, shrinkage and interlayer bond	Controls hydration rate, particle packing, water demand and structural build-up	Binder composition, replacement ratio, fineness and admixture compatibility
Fine aggregate grading and sand/binder ratio	Extrudability, filament stability and surface quality	Affects packing density, friction, pumpability and nozzle blockage risk	Maximum particle size, grading curve, sand/binder ratio and aggregate shape
Superplasticiser dosage	Pumpability and extrusion quality	Governs the balance between the flow needed for pumping and extrusion and the shape-holding capacity needed immediately after deposition	Type, dosage, addition sequence and compatibility with binder
Viscosity-modifying agents and fine fillers	Buildability and cohesion	Governs the balance between cohesion/structural build-up needed for buildability and the extrusion pressure required to pump the mixture	Type, dosage, mixing sequence and effect on yield stress and viscosity
Fibre type, length and dosage	Flexural strength, crack bridging, shrinkage and anisotropy	Fibre orientation is affected by extrusion and may not cross the critical interlayer crack plane	Fibre type, aspect ratio, volume fraction, surface condition and expected orientation
Nozzle geometry and layer height	Contact area, filament shape and interlayer quality	Determines filament cross-section, lateral spread, surface roughness and pressure at deposition	Nozzle size, shape, stand-off distance, layer height and print width
Printing speed and extrusion rate	Filament continuity and interlayer contact	Controls deposition pressure, filament tearing, surface finish and contact quality	Print speed, extrusion rate and synchronisation between material flow and movement
Interlayer time interval	Interlayer bond strength and cold-joint risk	Governs surface moisture loss, thixotropic rebuilding and early hydration of the lower layer	Time interval, ambient temperature, humidity and wind condition
Surface moisture condition	Wetting and chemical/mechanical integration between layers	Governs the degree of intimate contact and hydration continuity achievable across the interface	Surface moisture or qualitative surface condition at deposition
Specimen extraction and loading direction	Apparent strength and failure mode	Determines whether load crosses, follows or bypasses interlayer regions	Extraction direction, number of layers, loading direction and position within printed element

**Table 4:** Representative brick compressive-strength classes used for masonry unit classification.

Brick Grade	Compressive Strength (MPa)	Typical Application	Reference
M3.5	3.5	Wall construction	Huy et al. [53]
M5.0	5.0	Wall construction	Huy et al. [53]
M7.5	7.5	Wall construction requiring higher unit strength	Huy et al. [53]

**Table 5:** Representative mechanical-property observations for 3DPC and their structural interpretation.

Reported Property	Reported Response	Main Variable or Condition	Structural Interpretation	Reference
Compressive strength	75–102 MPa	3DPC printed using a 9 mm nozzle	High matrix strength can be achieved, but the result is linked to mixture and nozzle conditions.	Le et al. [1,2]
Compressive strength	About 79 MPa	Binder system containing CSA and Portland cement	Binder chemistry can strongly affect early and hardened strength development.	Khalil et al. [36]
Mechanical properties	Improved	Polypropylene fibre addition to 3DPC	Fibre addition may improve selected mechanical responses, but the effect depends on dosage, orientation and print direction.	Ye et al. [54]
Compressive and flexural strength	Improved	Partial cement replacement with glass powder	Replacement materials can enhance performance when particle packing and binder reactions are favourable.	Cuevas et al. [55]
Mechanical properties	Reduced	3DPC mixtures containing recycled glass	Recycled constituents may reduce performance if particle characteristics or bonding conditions are unfavourable.	Ting et al. [56]
Compressive strength	Increased	Decreased aggregate diameter	Smaller aggregate size can improve printability and matrix uniformity, but may also increase binder demand.	Zareiyan and Khoshnevis [57]
Mechanical and dimensional stability	Optimum response at selected fibre length and dosage	Polypropylene fibre length and volume fraction	Fibre efficiency is governed by aspect ratio, dosage and loading direction rather than fibre presence alone.	Şahin, Akgümüş and Mardani [14]
Strength and interlayer response	Direction-dependent behaviour	Loading direction and interlayer adhesion	Mechanical results must be interpreted according to print orientation and interlayer quality.	Şahin and Mardani [9,10]

**Table 6:** Existing standards and their relevance to structural qualification of 3DPC.

Standard	Main Scope	Transferable Value for 3DPC	Main Limitation for 3DPC	Required Modification or Additional Reporting
EN 1015-3 [106]	Consistence of fresh mortar by flow table	Provides a simple index of fresh mortar workability	Does not measure extrudability, buildability or shape retention after deposition	Add pumpability, extrudability, filament stability and open-time assessment
EN 1015-11 [107]	Flexural and compressive strength of hardened mortar	Provides baseline hardened mortar strength	Cast specimens do not represent printed layers or interlayer defects	Report printed or cast condition, specimen orientation and layer configuration
ASTM C270 [108]	Specification for mortar for unit masonry	Defines mortar proportioning and property requirements	Mortar requirements are not equivalent to printable mixture requirements	Add rheology, structural build-up and interlayer bond criteria
ASTM C780 [109]	Preconstruction and construction evaluation of mortars	Useful for field evaluation of mortar consistency and strength	Does not consider nozzle extrusion, deposition history or layer bonding	Add print-process quality control and restart-zone documentation
ASTM C91 [110]	Masonry cement specification	Provides physical and chemical requirements for masonry cement	Cement specification alone cannot predict printability or layer stability	Link binder characteristics to rheology, early stiffness and interlayer bond
ASTM C144 [111]	Aggregate for masonry mortar	Provides aggregate grading requirements	Masonry mortar grading does not address nozzle blockage and filament quality	Report maximum particle size, grading curve, particle shape and nozzle compatibility
ASTM C404 [112]	Aggregates for masonry grout	Defines grading of aggregates for grout	Grout flow is different from printable filament formation	Add extrusion pressure, segregation resistance and filament geometry control
ASTM C1329 [113]	Mortar cement specification	Provides requirements for mortar cement performance	Does not account for layer-wise deposition or structural build-up	Combine with rheological and early-age buildability tests
ASTM C1384 [114]	Admixtures for masonry mortars	Provides requirements for admixtures used in masonry mortar	Admixture performance in printing depends on shear history, open time and binder compatibility	Report admixture type, dosage, addition sequence, rheological response and interlayer bond
ASTM C1714 [115]	Preblended dry mortar mixes	Supports quality control of dry mortar mixtures	Dry mixture uniformity does not ensure printable behaviour	Add printability, storage stability, moisture control and extrusion performance
EN 1052-1 [116]	Compressive strength of masonry	Provides assemblage-level compression testing logic	Masonry prisms have mortar joints, not printed interlayers	Adapt prism concept to printed wall segments with defined layer orientation and extraction direction
EN 1052-2 [117]	Flexural strength of masonry	Recognises orientation-dependent flexural behaviour	Bed-joint and head-joint concepts do not directly match printed interfaces	Define flexural tests parallel, perpendicular and across printed layers
EN 1052-3 [118]	Initial shear strength of masonry	Provides a framework for joint-controlled shear behaviour	Printed interfaces are time-dependent and process-created	Include interlayer interval, surface condition, normal stress and print-path geometry
EN 1052-5 [119]	Bond strength by bond wrench method	Directly relevant to interface bond concepts	Bond wrench geometry is developed for unit–mortar joints	Develop adapted interlayer bond tests for printed filament interfaces
ASTM C1314 [120]	Compressive strength of masonry prisms	Distinguishes unit strength from assemblage behaviour	Printed coupons may not represent wall-scale filament continuity	Use printed prisms/wall segments with reported layer number, orientation and extraction location
ASTM E519/E519M [121]	Diagonal tension in masonry assemblages	Provides an assemblage-level shear/tension test concept	Diagonal cracking in 3DPC depends on print path, reinforcement and interlayer quality	Adapt for printed wall panels with defined print architecture and reinforcement detailing
Conventional concrete compression and flexural tests [122,123,124]	Basic concrete mechanical properties	Provide baseline compressive and flexural indices	May hide anisotropy if specimen orientation and layer history are ignored	Require direction-dependent testing and full reporting of print parameters
ACI Committee 564 activities [21]	Cementitious additive manufacturing guidance	Confirms the need for dedicated 3DPC guidance	Committee activity is not itself a complete design standard	Use as part of a broader standardisation framework
NIST measurement-science programme [22]	Performance-based standards for reinforced 3D printed concrete	Emphasises measurement science and structural performance	Does not replace project-specific qualification	Connect material tests, reinforcement strategy and performance-based validation
RILEM TC 304-ADC interlaboratory studies [23,24,25]	Mechanical test comparability for 3DPC	Provides evidence on the effect of extraction, curing, cold joints and testing procedure	Interlaboratory findings must still be translated into design provisions	Use to define reporting rules, specimen preparation and direction-dependent test protocols

**Table 7:** Proposed transfer matrix from masonry testing logic to 3DPC structural qualification.

Structural Issue	Masonry Testing Logic	Limitation in Direct Transfer to 3DPC	Recommended 3DPC Qualification Item
Compression	Unit and prism tests distinguish material and assemblage behaviour	Printed coupons may not represent wall-scale filament continuity, restarts or path changes	Report specimen orientation, layer height, nozzle size, extraction direction, age, curing and failure mode
Flexure	Bed-joint and head-joint directions are recognised	The critical tensile zone in 3DPC is process-created and time-dependent	Report print path, interlayer time interval, surface condition, extraction direction and failure plane
Shear	Mortar bond, normal stress, friction and reinforcement are considered	The shear plane may coincide with filament interfaces, cellular voids or poorly bonded layers	Report loading direction, interface geometry, normal stress, surface roughness and reinforcement continuity
Interlayer bond	Unit–mortar bond can be tested directly	Printed interlayers are influenced by moisture loss, thixotropy and deposition pressure	Use direct tension, splitting, shear and fracture tests across layers
Reinforcement	Reinforcement detailing and anchorage are treated as part of the load path	Reinforcement in 3DPC may be inserted, post-installed or discontinuous across layers	Report reinforcement type, location, anchorage, continuity across interfaces and grout or duct details
Seismic and cyclic response	Assemblage and wall tests are used for in-plane and out-of-plane actions	Full-scale 3DPC cyclic data remain limited and connection details dominate behaviour	Require wall-panel tests with realistic print paths, axial load, boundary conditions and cyclic protocol
Quality control	Units and mortar can be sampled separately	In 3DPC, material manufacture and placement occur simultaneously	Use inline geometry control, layer-dimension records, restart-zone records and cold-joint risk assessment
Design values	Design is based on assemblage behaviour and calibrated strength parameters	Direction-independent material values may be unsafe for printed structures	Assign direction-dependent or conservatively reduced design values until wall-scale validation is available

## Data Availability

No new data were created or analysed in this study. Data sharing is not applicable.

## Supplementary Materials

The following supporting information can be downloaded at: https://www.mdpi.com/article/10.3390/ma19173688/s1. Table S1: The complete set of studies included in this review.
