# Supplementary material for: Interface-Controlled Structural Behaviour of 3D Printed Concrete: Lessons from Masonry for Anisotropy, Print-Path Design and Standardisation
Source: Materials (Basel). 2026 Aug 30;19(17):3688. doi: 10.3390/ma19173688 (PMC13567561; doi:10.3390/ma19173688)
Supplement: Supplementary file 1 [file materials-19-03688-s001.zip › materials-4416113-supplementary.pdf]

Supplementary Table S1. Complete list of sources included in Table 1

The table lists every publication, review, guidance document and interlaboratory study used in Table 1.

References	Complete citation	Study type	Role in Table 1
[1]	Le, T.T.; Austin, S.A.; Lim, S.; Buswell, R.A.; Gibb, A.G.F.; Thorpe, T. Mix design and fresh properties for high-performance printing concrete. Materials and Structures 2012, 45, 1221–1232.	Experimental study	Mixture design, extrudability, buildability, workability and open time.
[2]	Le, T.T.; Austin, S.A.; Lim, S.; Buswell, R.A.; Law, R.; Gibb, A.G.F.; Thorpe, T. Hardened properties of high-performance printing concrete. Cement and Concrete Research 2012, 42, 558–566.	Experimental study	Hardened properties, anisotropy, interlayer tensile bond and drying shrinkage.
[3]	Buswell, R.A.; Soar, R.C.; Gibb, A.G.F.; Thorpe, A. Freeform construction: Mega-scale rapid manufacturing for construction. Automation in Construction 2007, 16, 224–231.	Conceptual / technology study	Early construction-scale additive-manufacturing framework.
[4]	Roussel, N. Rheological requirements for printable concretes. Cement and Concrete Research 2018, 112, 76–85.	Analytical / conceptual study	Rheological requirements for extrusion, deposition and buildability.
[5]	Wolfs, R.J.M.; Bos, F.P.; Salet, T.A.M. Early age mechanical behaviour of 3D printed concrete: Numerical modelling and experimental testing. Cement and Concrete Research 2018, 106, 103–116.	Experimental and numerical study	Early-age stiffness, strength, direct shear and construction-stage stability.
[6]	Panda, B.; Paul, S.C.; Tan, M.J. Anisotropic mechanical performance of 3D printed fiber reinforced sustainable construction material. Materials Letters 2017, 209, 146–149.	Experimental study	Direction-dependent mechanical performance.
[7]	Moini, M.; Olek, J.; Youngblood, J.P.; Magee, B.; Zavattieri, P.D. Additive manufacturing and performance of architectured cement-based materials. Advanced Materials 2018, 30, 1802123.	Experimental study	Architectured filaments, interfaces and fracture behaviour.
[8]	Şahin, H.G.; Mardani-Aghabaglou, A. Assessment of materials, design parameters and some properties of 3D printing concrete mixtures: A state-of-the-art review. Construction and Building Materials 2022, 316, 125865.	Review	Materials, mixture design, rheology and standardization context.
[9]	Şahin, H.G.; Mardani, A. Mechanical properties, durability performance and interlayer adhesion of 3DPC mixtures: A state-of-the-art review. Structural Concrete 2023, 24, 5481–5505.	Review	Mechanical anisotropy, durability and interlayer adhesion.
[10]	Şahin, H.G.; Mardani, A. How does rheological behaviour affect the interlayer-bonding strength of 3DPC mixtures? Journal of Adhesion Science and Technology 2024, 38, 1353–1377.	Focused review	Rheology–interlayer bond relationship.
[11]	Biricik, Ö.; Mardani, A. Parameters affecting thixotropic behavior of self compacting concrete and 3D printable concrete: A state-of-the-art review. Construction and Building Materials 2022, 339, 127688.	Review	Thixotropy, structural build-up and measurement methods.
[12]	Şahin, H.G.; Mardani, A.; Mardani, N. Performance requirements and optimum mix proportion of high-volume fly ash 3D printable concrete. Buildings 2024, 14, 2069.	Experimental study	High-volume fly ash mixture optimization and fresh-state requirements.
[13]	Şahin, H.G.; Mardani, A.; Beytekin, H.E. Effect of silica fume utilization on structural build-up, mechanical and dimensional stability performance of fiber-reinforced 3D printable concrete. Polymers 2024, 16, 556.	Experimental study	Silica fume, structural build-up, strength and shrinkage.
[14]	Şahin, H.G.; Akgümüş, F.E.; Mardani, A. Mechanical and rheological properties of fiber-reinforced 3D printable concrete in terms of fiber content and aspect ratio. Structural Concrete 2025, 26, 1597–1612.	Experimental study	Fibre length/content, rheology, anisotropy and shrinkage.
[15]	Şahin, H.G.; Akarsu Özenç, A.; Saka Dinç, Z.; Mardani, A.; Eren, S. Investigation of fresh and hardened properties of 3D printable concrete containing ozone-modified carbon fiber. Journal of Sustainable Cement-Based Materials 2025, 14, 534–548.	Experimental study	Ozone-modified carbon fibre, rheology and mechanical performance.
[16]	Akgümüş, F.E.; Şahin, H.G.; Mardani, A. Investigation of waste steel fiber usage rate and length change on some fresh state properties of 3D printable concrete mixtures. Buildings 2025, 15, 3731.	Experimental study	Waste tyre steel fibre, slag, printability and rheology.
[17]	Akgümüş, F.E.; Şahin, H.G.; İsafça Kaya, T.; Mardani, A. Combined effect of recycled tire steel fiber and blast furnace slag on the mechanical performance of 3D printable concrete. Buildings 2025, 15, 4564.	Experimental study	Mechanical, dimensional and interfacial performance of printable mixtures.
[20]	ACI Committee 564. 3-D Printing with Cementitious Materials. American Concrete Institute, Farmington Hills, MI, USA.	Guidance / standardization activity	3DPC-specific guidance and standardization context.
[21]	National Institute of Standards and Technology. Additive Manufacturing with Cement-Based Materials. NIST, Gaithersburg, MD, USA.	Guidance / measurement-science activity	Measurement science and standardization context.
[22]	Bos, F.; Menna, C.; Robens-Radermacher, A.; Wolfs, R.; Roussel, N.; Lombois-Burger, H.; Baz, B.; Weger, D.; Chen, Y.; et al. Mechanical properties of 3D printed concrete: A RILEM TC 304-ADC interlaboratory study—approach and main results. Materials and Structures 2025, 58, 183.	Interlaboratory study	Overall study design, comparability and principal findings.
[23]	Wolfs, R.; Versteege, J.; Santhanam, M.; Bhattacherjee, S.; Bos, F.; Robens-Radermacher, A.; Muthukrishnan, S.; Menna, C.; Ozturk, O.; Ozyurt, N.; et al. Mechanical properties of 3D printed concrete: A RILEM TC 304-ADC interlaboratory study—flexural and tensile strength. Materials and Structures 2025, 58, 182.	Interlaboratory study	Flexural, splitting tensile and uniaxial tensile testing.
[24]	Mechtcherine, V.; Muthukrishnan, S.; Robens-Radermacher, A.; Bos, F.; Menna, C.; Wolfs, R.; et al. Mechanical properties of 3D printed concrete: A RILEM TC 304-ADC interlaboratory study—compressive strength and modulus of elasticity. Materials and Structures 2025, 58, 181.	Interlaboratory study	Compressive strength and modulus of elasticity.
[25]	Bai, M.; Xiao, J.; Ding, T.; Yu, K. Interfacial bond properties between 3D printed engineered cementitious composite (ECC) and post-cast concrete. Cement and Concrete Composites 2025, 157, 105897.	Experimental study	Printed-ECC/post-cast-concrete interface, splitting tensile strength, shear strength, X-CT and SEM.
